# Calibrating the tree of vipers under the fossilized birth-death model

**DOI:** 10.1038/s41598-019-41290-2

**Published:** 2019-04-02

**Authors:** Jiří Šmíd, Krystal A. Tolley

**Affiliations:** 1 South African National Biodiversity Institute, Kirstenbosch Research Centre, Cape Town, South Africa; 2 Department of Botany and Zoology, University of Stellenbosch, Stellenbosch, Private Bag X1 South Africa; 3 Department of Zoology, National Museum, Cirkusová, 1740 Prague Czech Republic; 4 Department of Zoology, Faculty of Science, Charles University in Prague, Viničná 7, Prague, Czech Republic; 5 Centre for Ecological Genomics and Wildlife Conservation, Department of Zoology, University of Johannesburg, Auckland Park, 2000 Johannesburg, South Africa

## Abstract

Scaling evolutionary trees to time is essential for understanding the origins of clades. Recently developed methods allow including the entire fossil record known for the group of interest and eliminated the need for specifying prior distributions for node ages. Here we apply the fossilized birth-death (FBD) approach to reconstruct the diversification timeline of the viperines (subfamily Viperinae). Viperinae are an Old World snake subfamily comprising 102 species from 13 genera. The fossil record of vipers is fairly rich and well assignable to clades due to the unique vertebral and fang morphology. We use an unprecedented sampling of 83 modern species and 13 genetic markers in combination with 197 fossils representing 28 extinct taxa to reconstruct a time-calibrated phylogeny of the Viperinae. Our results suggest a late Eocene-early Oligocene origin with several diversification events following soon after the group’s establishment. The age estimates inferred with the FBD model correspond to those from previous studies that were based on node dating but FBD provides notably narrower credible intervals around the node ages. Viperines comprise two African and an Eurasian clade, but the ancestral origin of the subfamily is ambiguous. The most parsimonious scenarios require two transoceanic dispersals over the Tethys Sea during the Oligocene.

## Introduction

Scaling phylogenetic trees to time is one of the major challenges in evolutionary biology. Reliable estimates for the age of evolutionary events are essential for addressing a wide array of questions, such as deciphering micro- and macroevolutionary processes, identifying drivers of biodiversity patterns, or understanding the origins of life^1^.

The most widely used approach for dating trees is calibration at internal nodes (hereafter ‘node dating’)^2^, in which some nodes are *a priori* assigned ages based, for example, on the fossil record for that clade. Despite its wide use, there are drawbacks to this method. First of all, the fossil record is extremely fragmentary and the likelihood of recovering a fossil of a common ancestor of two lineages is extremely low^3^. Also, the earliest fossil representatives of newly divergent taxa might be virtually indistinguishable^4^. Fossils thus may provide information about the absolute minimum clade ages, but the maximum ages remain obscure^5^. For this reason, the age of the oldest known fossil is commonly used as the minimum hard bound, while the maximum clade age is given a soft bound with a specified prior distribution. Despite recent progress in prior distribution specification^6^ this task still remains challenging as the decision regarding optimal prior density is often problematic, especially for groups with a poor fossil record^2^. Recently, alternative methods that use fossils to calibrate trees have been developed. One is the fossilized birth-death model (FBD)^7^ that uses the entire fossil record rather than just the oldest fossil for a given node. Similar to node dating, FBD requires some prior knowledge about the phylogenetic affinities of the fossils, but contrary to node dating, no prior calibration densities of the node ages need to be specified as fossils are assigned to branches or clades and not nodes^7,8^. While node dating requires the user to parameterize a prior density for each calibrated node, the FBD model assumes random sampling of extant and fossil species, constant speciation and extinction rates, and prior information on these FBD model parameters: speciation rate (*λ*), extinction rate (*μ*), fossil recovery rate (*ψ*), and proportion of sampled extant species (*ρ*).

Here, we apply the FBD approach to reconstruct the timeline of viperine diversification. Viperines (subfamily Viperinae) are an Old World radiation distributed through Africa (excluding Madagascar), Arabia, Europe and Asia^9^. The subfamily comprises 102 species in 13 genera. The sister relationships within Viperidae (Viperinae to Azemiopinae + Crotalinae) and its monophyly are well supported by available genetic evidence^10,11^, although this does not reflect relationships based on analyses of morphological data. Instead, morphology shows that the genus *Causus* possesses features similar to *Azemiops* or other colubroid snakes^12^. Latest phylogenies based on hundreds of phenotypic characters reconstructed *Causus* as sister to the Viperinae + Crotalinae^13,14^. Furthermore, the incongruence in the phylogenetic position of *Causus* is also obvious from genetic-based phylogenies. While early studies placed it as sister to the Viperinae^15,16^, more recent analyses with broader taxon and gene sampling placed it within the radiation of viperines. However, the placement within Viperinae has been inconsistent and the genus has been placed as sister to *Echis* + *Cerastes*^17^, sister to *Atheris*^18^, sister to *Echis*^10,19,20^, sister to six Eurasian genera^21^, and sister to *Proatheris*^11,22,23^. As a result of these topological inconsistencies, the geographic origin of vipers has not been resolved, although both genetic and fossil evidence suggest a non-European origin^17,24^.

**Figure 1:**
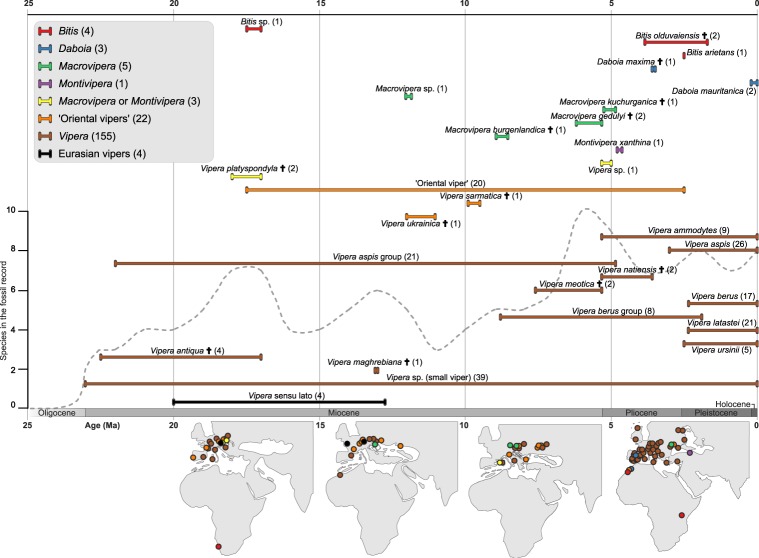
Temporal and spatial distribution of the viperine fossil record. All fossil specimens that belong to a species or species group are shown as one horizontal bar. Bars are colored by genera or higher clades as denoted in the upper left panel. Black crosses denote extinct species. ‘Oriental vipers’ represents an assemblage of generally large-bodied species belonging to either of the modern genera *Daboia*, *Macrovipera*, or *Montivipera*. Eurasian vipers are a clade of the genera *Vipera*, *Daboia*, *Montivipera*, and *Macrovipera*. Numbers in parentheses show the number of individual fossils for each species and genus. The dashed grey line shows the number of species in the fossil record through time. The maps show approximate locations of fossils reported from five-million-year timeframes from 20 Ma to the present. Note that not all fossils are plotted for the lack of space and one locality for an ‘Oriental viper’ in eastern Russia is not shown in the map.

Vipers are well represented in the fossil record, although their remains are fragmentary and usually consist of isolated vertebrae, cranial segments and fangs^24,25^. The oldest viperine fossil is a central European *Vipera antiqua*, which is dated to the early Miocene, ca. 22.5 million years ago (Ma, Fig. 1)^26^. Osteological features preserved in viperid fossils generally permit identification, although certain characters are rather homogeneous and shared across different species. This conserved morphology has led to the establishment of species groups, which contain taxa that are osteologically similar^12,27^. For example, the small European vipers of the genus *Vipera* form two morphological groups (*V. berus* and *V. aspis* groups) that differ in vertebral and cranial morphology, but differentiating species within these groups is nearly impossible^24^. Likewise, fossil remains of large-bodied species have traditionally been clustered in a group of ‘Oriental vipers’^28^. However, monophyly of the ‘Oriental vipers’ is questionable as it has not been confirmed either by morphological analyses^29^, immunological comparisons^30^ or multilocus phylogenetic analyses^10,11,22^.

**Figure 2:**
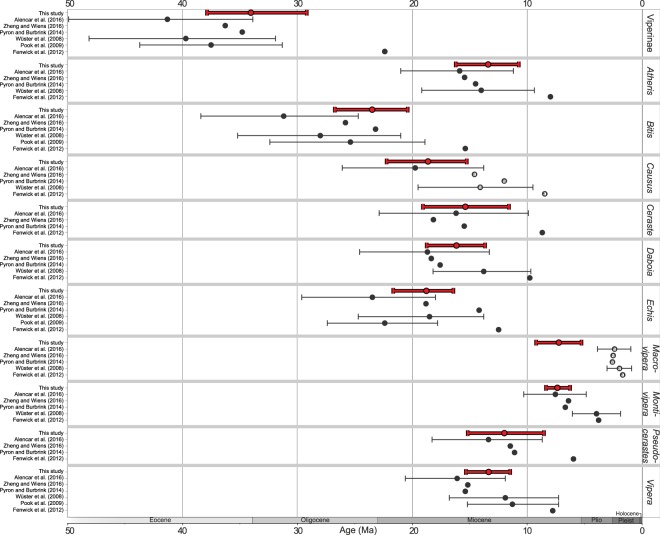
Comparison of crown ages of the viperines and their genera as estimated in this (in red) and previous studies. Circles represent mean age estimates and bars indicate their 95% HPD credible intervals (not available for some studies). Circles marked with an ‘a’ indicate that the oldest species was missing in the analysis. The monotypic genera *Eristicophis* and *Proatheris* are not shown as they only have stem ages. Exact age estimates are given in detail in Supplementary Table S4.

Several studies have estimated the time-calibrated phylogeny for viperines, all of which used the node dating approach with calibration points almost exclusively outside the viperines. The sole exception was fossil evidence for the initial radiation of the Eurasian vipers used by some authors^17,18^, but without referring to an actual fossil. Most agree on the origin of the crown Viperinae to be dated between the middle Eocene and early Miocene (ca. 34–42 Ma with confidence intervals ranging between 32 and 50 Ma; Fig. 2)^11,13,17–19,22^ (but see^31^).

In this study, we reconstruct the phylogeny of Viperinae using an unprecedented taxon sampling of 83 modern species (out of 102) and 13 genetic markers in combination with their entire known fossil record. This new assessment allowed for a comparison of the FBD approach to previously published results based on node dating. Additionally, we use the new phylogeny for ancestral area reconstructions to examine the origin and speciation history of viperines.

## Materials and Methods

### Sampling of Modern Species

Of the 102 species of Viperinae, there were sequence data available for 79 species on GenBank, which was downloaded to collate a dataset of 13 genes, six mitochondrial and seven nuclear (Supplementary Table S1). The number of available sequences ranged from 1 to 12 per species (with the mean of 4.7). Additionally, we generated a total of 46 new DNA sequences of seven markers for 23 species of the genera *Bitis* and *Causus* (Supplementary Table S2). The mitochondrial dataset was 59% complete, the nuclear one was 22% complete. The best sampled mitochondrial markers were cytochrome *b* (available for 77 species) and 16S rRNA (available for 62 species), the nuclear ones were PRLR (available for 27 species) and UBN1 (available for 23 species). *Eristicophis*, *Proatheris*, and *Pseudocerastes* only had mitochondrial markers available. The final data matrix comprised 422 sequences representing 83 modern species.

### Sampling of Fossil Species

Data on the viper fossils, their age ranges, and geographic origins were obtained from the regularly updated fosFARbase^32^ that contained 302 unique fossils of the Viperinae. We pruned the list by removing duplicate, dubious, and undetermined records. As dubious we considered fossils with doubtful phylogenetic placement (e.g. *Vipera aegertica*, *V. kargii*)^24^ or those originally assigned to viperines but later redetermined (e.g. *Vipera* sp. from Oggenhausen, Germany)^24,33,34^. Fossils were assigned to species or alternatively to higher taxonomic groups when quality of the material prevented assignment to a particular species. We verified all records with primary and secondary literature for a more accurate taxonomic assignment (details are given in Supplementary Table S3). The final dataset contained 197 unique fossil records (Fig. 1, Supplementary Table S3). The species groups of *Vipera* and the ‘Oriental vipers’ as used in the paleontological literature must be treated with caution as they might not represent clades. For example, the *Vipera aspis* group found in the paleontological literature is in fact a paraphyletic assemblage composed of two clades, one of *V. aspis*, *V. latastei*, and *V. monticola* and the other of *V. ammodytes* and *V. transcaucasiana*. Under the FBD model, these fossils may be assigned to deeper nodes of the tree, although this increases uncertainty in the placement of the fossil^7^. There were five recent viper genera represented in the fossil material: *Bitis* (4 records), *Daboia* (3), *Macrovipera* (5), *Montivipera* (1), *Vipera* (155). Additionally, 29 records could not be assigned to a genus and were assigned to higher groups: the ‘Oriental vipers’ (22 records), *Macrovipera* or *Montivipera* (3), the Eurasian genera *Vipera*, *Daboia*, *Montivipera*, *Macrovipera* (4) (Fig. 1).

### Phylogenetic Analyses and FBD Dating

All sequences were checked and edited in Geneious 11.1.3 (www.geneious.com). We aligned each marker independently using MAFFT 7^35^ and the ‘auto’ settings for all genes. We used Gblocks^36^ to remove poorly alignable sites of the rRNA mtDNA genes. Fifteen outgroup species were included in the dataset, six from the closest subfamilies Crotalinae (5 species) and Azemiopinae (1 species) and nine from other snake families (Supplementary Table S1). The concatenated alignment had a length of 10418 bp (Supplementary Table S2). The dataset was partitioned by gene, with the best models of nucleotide evolution estimated using PartitionFinder (PF)^37^. To avoid over-parameterization we used the HKY instead of the GTR model for all partitions, with the among-site rate variation parameter (+Γ) included for the 12S, 16S, cox1, cytb, nd2, nd4, cmos, nt3, and prlr partitions as identified by PF. Invariant sites (+I) were excluded, as this is accounted for by the +Γ parameter^38^.

All analyses were run with BEAST 2.5.1^39^ at the CIPRES Science Gateway^40^, using the Sampled Ancestors (SA 2.0) package^41^. Likelihood ratio tests (LRT) implemented in MEGA6^42^ rejected the clock-like models of evolution for all partitions except the cox1; we therefore used a strict-clock prior for the cox1 and relaxed clock lognormal priors for all other partitions. We used the FBD model as the tree prior. The parameters of the FBD model are reparameterized as follows: diversification rate *d* = *λ* – *μ*; turnover rate *r* = *μ*/*λ*; fossil sampling proportion *s* = *ψ*/(*μ* + *ψ*)^7^. We assumed an exponential prior for *d* with the mean of 2.0 (lineages per million years). This broad prior distribution (5% and 95% quantiles being, respectively, 0.103 and 5.99) reflected the difficulty of predicting the speciation and extinction rates *a priori*. For the *s* and *r* parameters we used beta prior distributions with shape parameters *α* = 2.0 and *β* = 2.0. Beta distribution ranges between 0 and 1 and such shape parameters *α* and *β* produced a convex-shaped distribution with the highest probability at 0.5. This shape was chosen because extremely high (close to 1.0) or low (close to 0.0) values for these two parameters could not be ruled out, but are not probable. The *ρ* parameter was given a value of 0.81 as 83 of 102 modern viperines were included in the analysis. We assumed a lognormal prior distribution for the rate of the mtDNA genes (parameter ucld.mean; mean = 0.3 [substitutions per site per million years], SD = 1.0) and an exponential prior distribution for the nDNA genes (mean = 0.05). Rate heterogeneity among lineages (parameter ucld.stdev) was assumed to have an exponential prior distribution for all partitions (mean = 1.0). The clock rate of cox1 was assumed to have a lognormal prior distribution (mean = 1.0 [substitutions per site per million years], SD = 1.25).

The set of 197 fossil records represented 28 different species or species groups, which were added to the dataset with sequences coded as missing data. For fossil species known only from a single record (11 species in total), the stratigraphic age was represented by the minimum and maximum age estimate for the particular record. For fossils with more than one record, the youngest and oldest records were used as the age limits. Means of the stratigraphic ages were used as the initial values for all records. Because all fossils in the dataset were crown fossils we conditioned the FBD model on the root. We specified 21 constraints on the phylogeny to indicate the phylogenetic placement of the fossils (Supplementary Table S3). They may be found in the supplementary nexus alignment file. The *Vipera aspis* group and ‘Oriental vipers’ were defined without enforcing their monophyly because the two groups may not be monophyletic. We ran three independent runs each for 5 × 10^8^ generations with parameters logged every 25,000 generations. Posterior trace plots, stationarity, convergence and effective sample sizes (ESS) were inspected in Tracer 1.5^43^. We discarded the first 10% of sampled trees from each run as burn-in based on inspection of likelihood trace plots and combined the tree files using LogCombiner. Because the phylogenetic placement of the fossil samples was entirely a result of the prior we removed them from the posterior trees and then summarized the resulting trees with a maximum clade credibility (MCC) tree with mean node heights using TreeAnnotator.

### Ancestral Area Reconstruction

To infer geographic origins of viperines, we performed likelihood-based analyses in BioGeoBEARS^44^. Given the distribution pattern of modern viperines and geological history of the Old World, we defined four discrete biogeographic areas: Africa, Arabia, Asia, and Europe. Tree tips were assigned to one or more areas based on their current distributions^9^ and the outgroup was removed prior to the analysis. Using the viperine-only tree allowed reconstructing the ancestral range for the subfamily, but without accounting for deeper phylogenetic and biogeographic context. Therefore, to gauge the effect of outgroup on the biogeographic reconstruction we ran an additional analysis on a tree that besides viperines included all sequenced species of their sister clade - the subfamilies Azemiopinae (1 sp.; 50% of species included) and Crotalinae (198 spp.; 82% of species included). Sequence data for the additional subfamilies were obtained from GenBank and the dataset comprised 282 species (Supplementary Table S1). To generate the tree we used the same settings as described above for the FBD analysis except we assumed a birth-death model for the tree prior with a beta prior distribution (*α* = 2.0, *β* = 2.0) for the relative death rate and a uniform prior (0, 1000) for the birth rate. We ran the analysis four times for 2 × 10^8^ generations, logging parameters and trees every 25,000 generations, and 10% of trees were discarded as burn-in. The MCC tree was then used as the input tree.

For both trees, we applied all biogeographic analyses available in BioGeoBEARS (dispersal–extinction–cladogenesis – DEC; dispersal–vicariance analysis – DIVA; BayArea), all with and without the parameter + J employed, which allows for founder-event speciation^44^. The fit of the models was assessed by the sample-size corrected Akaike Information Criterion (AICc).

## Results

### Phylogenetic Analyses and FBD Dating

The three MCMC runs converged after 5% of the 5 × 10^8^ generations. Most parameters had adequate ESS, except for the age parameter of the most recent common ancestors of *Bitis arietans* and its fossil sample, and of *B. arietans* and *B. olduvaiensis* (ESS = 50 and 64, respectively). These low values were likely caused by the long branch leading to *B. arietans* (23.5 Ma) and the uncertainty associated with exact placement of the two fossils along this branch. The mean estimate of the net diversification rate was 0.072 lineages per million years with the 95% highest posterior density (HPD) interval ranging between 0.052–0.09. The mean estimate of the sampled ancestors was 22.7 individuals (HPD: 19–25), indicating that of the 28 fossils most were direct ancestors of other fossils or modern taxa. Evolutionary rates estimated for the individual genes are given in Supplementary Table S2.

**Figure 3:**
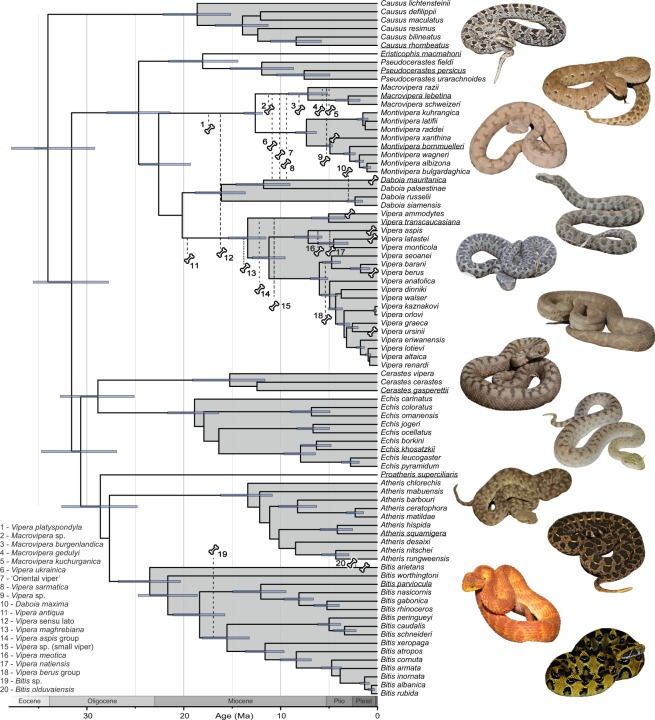
Maximum clade credibility tree for the modern viperines. The tree was calibrated with 197 fossils representing 28 species, whose phylogenetic positions are indicated by bone symbols; the numbered ones were assigned to a branch or a tree portion as indicated by the stippled lines and are of fossils listed in lower left. Unnumbered bone symbols are fossils of species known from the present day. Blue bars indicate 95% HPD intervals of the age estimates and are shown only for supported nodes with pp ≥ 0.95. Genera are highlighted in grey except the monotypic *Eristicophis* and *Proatheris*. Outgroup species are not shown. Specimens depicted on the right are of the species underlined and ordered as in the tree. Specimens are not to scale. Photographs are courtesy and are published with permission of T. Mazuch (*B. parviocula*, *D. mauritanica*), D. Jandzik (*E. macmahoni*), S. Carranza (*C. gasperettii*, *P. persicus*), D. Hegner (*A. squamigera*, *P. superciliaris*); otherwise JŠ. The tree with all age estimate and pp values is available in a nexus format in the supplementary materials.

Monophyly of all genera for which monophyly was not enforced was highly supported (posterior probability [pp] = 1.0). The genus *Causus* was placed as sister to all other viperine genera (pp = 0.98; Fig. 3). The remaining genera formed two well supported clades, one consisting of *Cerastes*, *Echis*, *Proatheris*, *Atheris*, and *Bitis* (pp = 0.99), the other of *Eristicophis*, *Pseudocerastes*, *Daboia*, *Macrovipera*, *Montivipera*, and *Vipera* (pp = 0.99). Phylogenetic relationships among the genera were generally well resolved, *Cerastes* was sister to *Echis* (pp = 1.0) and these two were sister to *Proatheris*, *Atheris*, and *Bitis* (pp = 0.98). Relationships between these three genera were not resolved. In the other clade, *Eristicophis* was supported as sister to *Pseudocerastes* (pp = 1.0), which were together sister to a clade of the Eurasian genera *Vipera*, *Daboia*, *Montivipera*, *Macrovipera*. Within the latter clade, *Montivipera* and *Macrovipera* were supported as sister lineages (pp = 1.0), but otherwise the relationships remained unresolved due to low support (pp = 0.54). The tree with all age estimates and support values is shown in Supplementary Fig. S1 and is supplied as a nexus file in the electronic Supplementary Materials.

The mean crown age of the Viperinae was estimated to 34.0 Ma (HPD: 29.2–37.8). Mean crown age estimates for the individual genera were mostly within the Miocene, ranging from 7.2 Ma in *Macrovipera* to 23.5 Ma in *Bitis* (Figs 2 and 3). Stem and crown age estimates for all genera and their HPD confidence intervals are given in Supplementary Table S4.

### Ancestral Area Reconstruction

**Figure 4:**
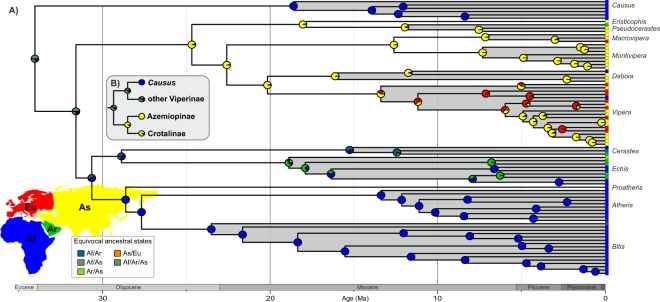
(**A**) Ancestral area reconstruction of the viperines. Genera are shaded, and species are in the same order as in Fig. 3. The four biogeographic areas defined for the analyses are shown in the map. Probabilities of the regions to be ancestral to a clade are shown as pie charts on the given nodes. (**B**) Results of the alternative biogeographic analysis that included all members of the Azemiopinae and Crotalinae. A complete tree with all 282 tips is shown in Supplementary Fig. S2.

In all analyses, models with the + J parameter were favored by LRT. The DEC + J was identified as the best-fitting model for both trees analyzed (Supplementary Table S5). According to the reconstructions (Fig. 4), the genera *Causus*, *Atheris*, *Bitis*, and *Proatheris* are of African origin, *Daboia*, *Eristicophis*, *Pseudocerastes*, *Macrovipera*, *Montivipera* are of Asian origin, *Echis* is most likely of Arabian origin (marginal probability for an Arabian origin 57%), *Vipera* is Eurasian (61% for Asia, 22% for Europe, 16% for unresolved Eurasian), and Cerastes is of an unresolved African or Arabian origin (61% for either Africa or Arabia). At deeper phylogenetic levels, the clade of *Eristicophis, Pseudocerastes*, *Macrovipera, Montivipera*, *Daboia*, and *Vipera* is clearly Asian in origin (96% for an Asian origin). The clade of *Proatheris*, *Atheris*, and *Bitis* is unequivocally African in origin (98%), and the clade of these three genera together with *Echis* and *Cerastes* originated either in Afro-Arabia or Africa (45% for Afro-Arabia, 39% for Africa). The geographic origin of all viperines except *Causus* was not resolved, with Afro-Arabia or Asia being the most supported (49%), followed by the option of either African or Asian origin (21%). Likewise, the biogeographic origin of the entire subfamily Viperinae remained unclear, with 50% support for Afro-Arabia or Asia, 25% for Africa or Asia, and 16% for Africa alone.

The alternative uncalibrated phylogenetic analysis that included viperines and all Azemiopinae and Crotalinae species resulted in a topology mostly corresponding to that of the FBD analysis. The difference was in the position of *Daboia* that was sister to the clade of *Macrovipera* and *Montivipera*, as opposed to the FBD analysis in which it was sister to *Vipera*. However, neither of the topologies was convincingly supported (pp = 0.73 and 0.54, respectively). The biogeographic reconstruction based on this 282-tip tree provided results similar to that of the Viperinae tree alone (Supplementary Fig. S2). While the origin of the individual viperine genera and deeper nodes within the subfamily remained well resolved (the Asian and Afro-Arabian clades were present), the biogeographic origin of the crown Viperinae and the node internal to the *Causus* clade remained obscure. The basal node of Viperinae was of an unresolved African/Arabian/Asian origin (marginal probability 49%) or African/Asian origin (32%), the node of viperines without *Causus* was also African/Arabian/Asian in origin (50%) or African/Asian (31%). Of a note is the reconstructed biogeographic origin of the entire family Viperidae, i.e. the clade containing the Viperinae, Azemiopinae and Crotalinae, which originated in Afro-Arabia or Asian (Africa/Arabia/Asia 49%, Africa/Asia 35%). The common ancestor of Azemiopinae and Crotalinae originated in Asia (95%).

## Discussion

In this study, we take a novel approach to reconstruct the evolutionary history of viperines. The FBD model used to calibrate the tree allowed integration of the actual fossil record of the group instead of relying on external calibration points, as previous studies using node dating have done^11,17–19,22,31,45^. We show that diversification within viperines began at the Eocene/Oligocene boundary (ca. 34 Ma). Stem lineages of most genera were established prior to the Miocene, and their crown ages were generally placed in the early to middle Miocene, with *Bitis* being the oldest genus that began to diversify at the end of the Oligocene (23.5 Ma). The FBD analysis yielded a slightly younger age estimate for the crown Viperinae compared to previous studies, although the credible intervals show considerable overlap (Fig. 2). Our age estimates for the individual genera are essentially congruent with dates from earlier works that used node dating^11,17–19,22,45^, with the exception of the study by Fenwick, *et al*.^31^, who estimated younger ages for most genera in spite of using calibration points similar to some of the other studies.

We show that the age of the genus *Macrovipera* is older than previously thought. This is, however, not a result of the FBD approach, but due to a recent taxonomic change in the genus^46^. *Macrovipera* and *Vipera* have of all the genera the richest fossil record that spans back before the modern species started diversifying. The earliest record of *Macrovipera* dates back to the middle Miocene (ca. 12 Ma; Fig. 1)^47^, while the modern species diverged relatively recently in the late Miocene (7.2 Ma). *Vipera* started appearing in the fossil record in the early Miocene (ca. 22.5 Ma)^24,26^, but its crown diversification is nearly ten million years younger; it dates to the middle Miocene (13.4 Ma). While *Vipera* has radiated into 24 today’s species, the diversity of *Macrovipera* has not changed significantly since the late Miocene, indicating low net diversification rate of the genus. Therefore, accounting for the fossil record is key not only for estimating the age of modern viperine taxa, but it is also crucial for assessing their past diversity.

The FBD model is being increasingly used for calibrating phylogenies and many studies have shown that node ages estimated under the FBD model rarely match those resulting from node dating analyses^7,48–50^. The congruence of our age estimates with previous studies that were based on node dating is thus fairly unique. It suggests that the calibration estimates in the squamate phylogeny are relatively stable (at least in the viper clade), and that most previous studies have produced concordant node age estimates, regardless of the uncertainty around the fossil calibration points. Compared to previously published time-trees of viperines, the current analysis is an improvement to the credible intervals around node ages, which are now notably smaller compared to those based on node dating. This may be due to the relatively high number of fossil species in our dataset (25% of taxa in the dataset are fossils) as it has been shown that higher proportion of fossils in the dataset results in shorter credible intervals^7,48^. Potentially relevant results have been recently provided by Harrington and Reeder^51^, who utilized FBD to calibrate a squamate-wide phylogeny. However, their taxon sampling of snakes, and vipers in particular, was not dense enough to permit direct comparison with our results.

Despite the relatively rich fossil record of Viperinae with nearly 200 unique fossils, only one of these has been used as a calibration point in node dating analyses^17,18^. This is most likely due to the difficulty in assigning viper fossils to nodes because of uncertainty regarding their ancestor-descendant relationships^24^. The FBD model overcomes this problem by allowing the use of the entire fossil record even if some fossils lack accurate taxonomic determination. This eliminates the need for using only the oldest fossil for a given node and the assignment of prior densities placed on the node ages, which is a major step forward in estimating divergence times. Moreover, FBD is robust against disproportional sampling^7,48^, which makes it applicable even for clades with an unevenly distributed fossil record, as is the case of viperines. Although the tree topology is not required to be fixed^41^, certain knowledge of the relationships among the taxa studied is still needed to be able to assign the fossils to existing clades. This limitation is overcome by the total evidence approach^52,53^, which can also use the FBD model for the tree prior. This approach uses the entire fossil record, but requires morphological characters to be scored for both fossil and modern species. Such data matrices may, however, not be readily available or obtainable for many groups of organisms. Hence, of the methods developed to calibrate phylogenetic trees, the FBD has, in our view, the biggest potential to substitute the currently prevailing node dating approach.

Although we have generated the most complete phylogeny of the viperines to date, the ancestral reconstructions of the geographic origin of the group were not conclusive. Our analyses show that the crown Viperinae are of an African, Arabian or Asian origin (or combinations therein). Despite this, we can now make some valuable inferences that have not been possible to date. Firstly, a solely Arabian origin can be ruled out because Arabia was part of the African continent in the early Oligocene when viperines began to diversify^54^. Likewise, Europe and Asia were a single continent throughout the history of the viper family, although Europe was periodically fragmented into a series of islands by the expanding Paratethys Sea^55,56^. This narrows down the possible place of origin to two landmasses: Afro-Arabia and Eurasia. Eurasia was part of Laurasia while Afro-Arabia is Gondwanan in origin, and the two landmasses remained widely separated by the Tethys Sea until their collision in the early Miocene^57^. Thus, the origin of viperines would have to be either one or the other landmass and their current distribution would then necessitate sub-aerial dispersal across the Tethys Sea.

**Figure 5:**
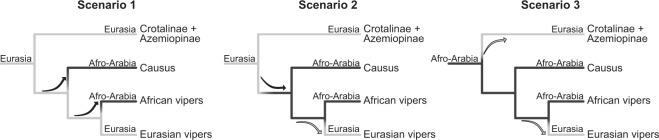
Plausible scenarios of the biogeographic history of the family Viperidae. Black and grey branches correspond to the Afro-Arabian and Eurasian distributions, respectively. Black arrows indicate a trans-Tethys dispersal from Eurasia to Afro-Arabia, grey arrows from Afro-Arabia to Eurasia.

While the origin of the crown Viperinae was not resolved in any of the analysis, the clade of Crotalinae and Azemiopinae is clearly Asian (Supplementary Fig. S2)^17,58^. Given this, and the viperine topology recovered here, three equally parsimonious but mutually exclusive scenarios could explain the dispersal history of vipers (Fig. 5). The family Viperidae could have originated in Eurasia and dispersed to Afro-Arabia in two consecutive steps ca. 34 and 32 Ma across the Tethys Sea. The second scenario also assumes a Eurasian origin, but with an over-sea dispersal to Afro-Arabia followed by re-colonization of Eurasia between 25–32 Ma. The third scenario is that Viperidae originated in Afro-Arabia and dispersed to Eurasia twice across the Tethys, first as the common ancestor of the Crotalinae and Azemiopinae, then as the ancestor of the Eurasian genera *Eristicophis*, *Pseudocerastes*, *Daboia*, *Macrovipera*, *Montivipera*, and *Vipera*. All three scenarios assume two independent trans-Tethys dispersal events, each followed by a substantial *in situ* radiation. Previous biogeographic analysis supports the second scenario^17^, although that study was based on smaller taxon and gene sampling and a tree topology that differed slightly from the present study (*Causus* sister to *Echis* + *Cerastes*). A non-European origin for Viperinae was also suggested based on the fossil evidence^24^. Given the current evidence available, it is not possible to provide a more definitive answer to this biogeographic conundrum other than the three equally parsimonious scenarios.

The viperine fossil record shows a clear pattern of increasing diversity towards the middle Miocene and then towards the recent past (Pliocene, Pleistocene; Fig. 1). The Miocene burst corresponds temporarily to the Miocene Climatic Optimum (MCO; ca. 14–18 Ma), an era of increased global temperatures relative to the long-term average^59^. This warm pulse could have provided suitable conditions for ectothermic vertebrates to thrive and diversify in regions that are currently less favorable to them, such as central and eastern Europe^60^. MCO was followed by global cooling, which could have been a driver of extinction especially for high-latitude faunas^61,62^, as evidenced by some viper extinctions at that time (e.g. *V. sarmatica*, *V. ukrainica*, *M. burgenlandica*). Indeed, it appears that the European reptile community was much richer prior to this cooling period, as many clades previously present in Europe shifted toward tropical or subtropical distributions (e.g. Boidae, Chamaeleonidae, Elapidae, Lamprophiidae)^63–65^.

In conclusion, our genetic dataset of modern viperines is the most comprehensive to date in terms of taxon and gene sampling. The phylogeny was well supported in most relevant nodes including the position of *Causus*, a genus whose position has proven difficult to infer previously. Our improved dataset and the inclusion of fossils in the dating analyses provide more confident interpretations regarding the evolutionary history of this group. It appears that Viperinae started diversifying at the Eocene/Oligocene boundary and the radiation of most genera took place in the Miocene. The late-Miocene cooling probably resulted in extinctions at high latitudes, with some taxa shifting to lower latitudes. While the biogeographic origin of the subfamily Viperinae remains unclear, we propose scenarios that explain their current distribution pattern, all of which require two transoceanic dispersals.

## Supplementary information

Supplementary Materials

Additional Dataset 1
