# Supplementary material for: Calibrating the tree of vipers under the fossilized birth-death model
Source: Sci Rep. 2019 Apr 2;9:5510. doi: 10.1038/s41598-019-41290-2 (PMC6445296; doi:10.1038/s41598-019-41290-2)
Supplement: Supplementary file 1 — Supplementary Materials [file 41598_2019_41290_MOESM1_ESM.pdf]

Calibrating the tree of vipers under the fossilized birth-death model

Jiří Šmíd, Krystal A. Tolley

SUPPLEMENTARY MATERIALS

Contents:

Supplementary Figure S1 2
Supplementary Figure S2 3
Supplementary Table S1 4
Supplementary Table S2 12
Supplementary Table S3 13
Supplementary Table S4 33
Supplementary Table S5 34
References for Supplementary Tables 35

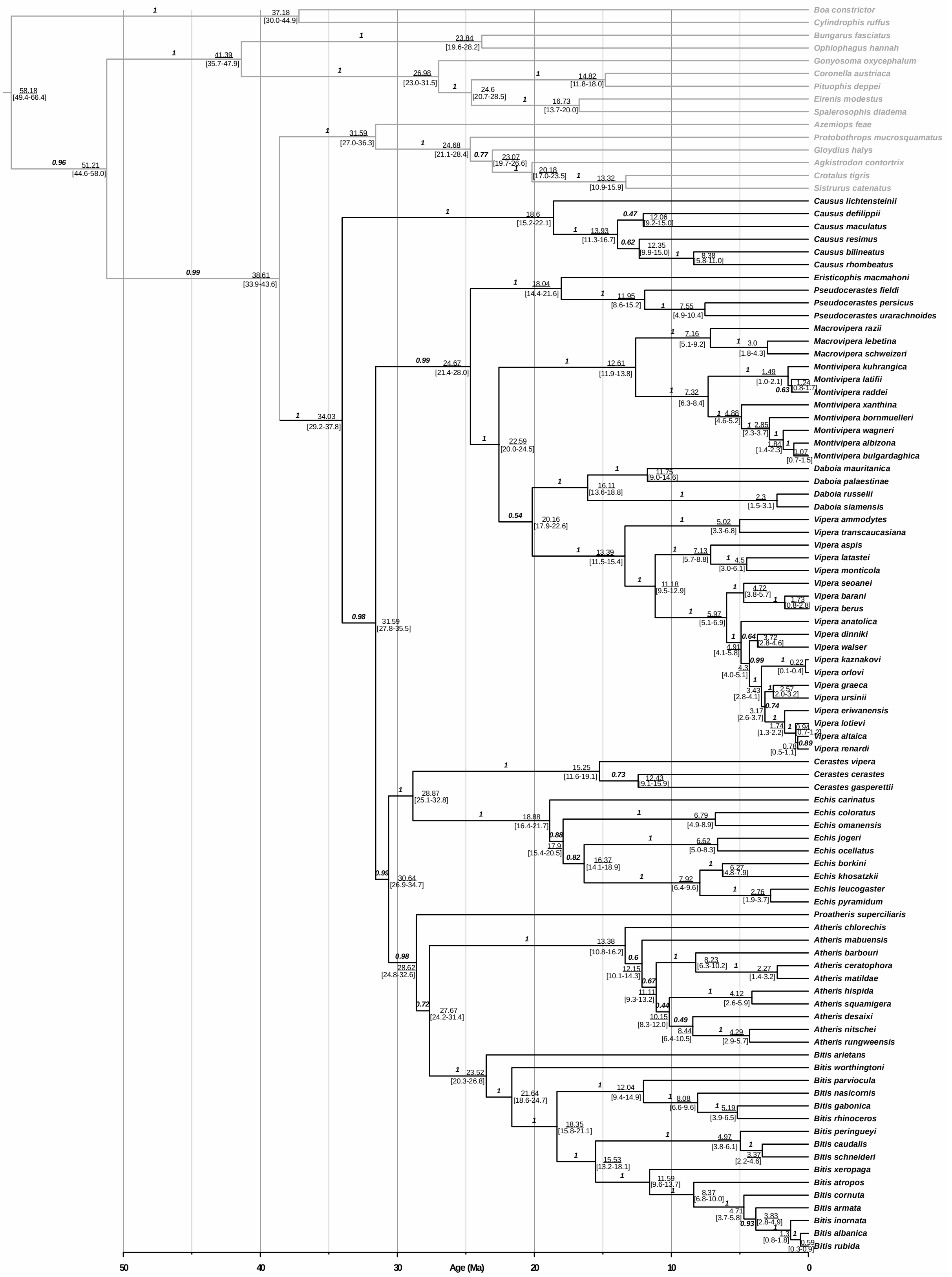

Supplementary Figure S1. Phylogeny of the viperines (black part of the tree) calibrated using 197 fossils of 28 species and the FBD model. The tree is a maximum clade credibility tree with mean node heights. Fossil tips have been removed from the tree because their phylogenetic placement was entirely a result of the prior. Posterior probability values are in italics and bold, mean node age estimates are underscored and their 95% HPD intervals are in brackets.

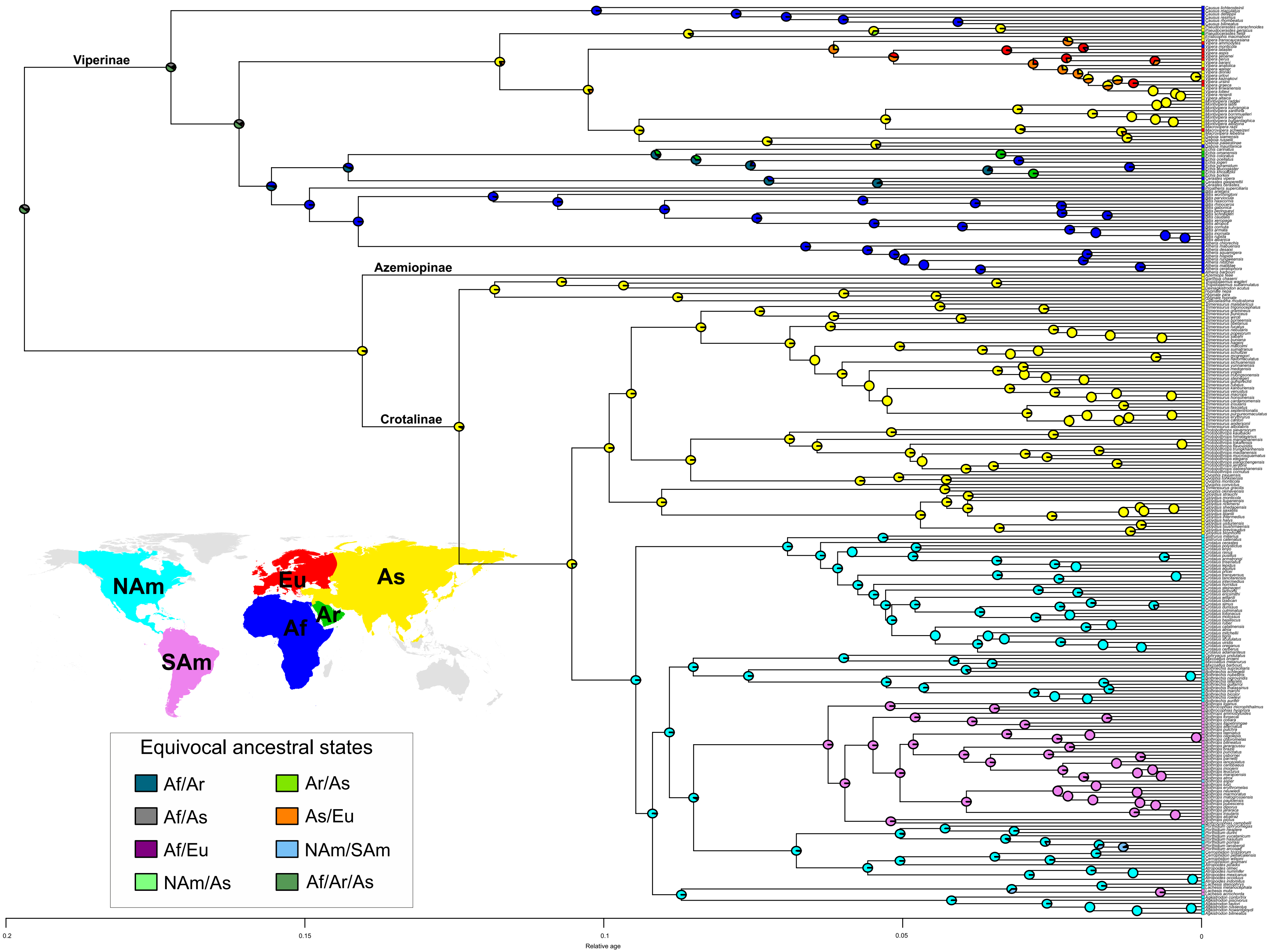

Supplementary Figure S2. Ancestral area reconstruction based on the tree of Viperinae and their closest relatives, Azemiopinae and Crotalinae. Note that the analysis was not time-calibrated, the scale of the tree is thus in relative time units.

Supplementary Table S1. Sequences used for the phylogenetic analyses. Outgroup species marked with an asterisk in the 'Sample' field were used as the outgroup in the FBD analysis.

					mtDNA						nDNA						
**Clade**	**Genus**	**Species**	**Sample**	**Genes**	**12S**	**16S**	**COI**	**CYTB**	**ND2**	**ND4**	**BDNF**	**CMOS**	**JUN**	**NT3**	**PRLR**	**RAG1**	**UBN1**
Viperinae	*Atheris*	*barbouri*	MTSN7311	3		AJ275739		AJ275686		MF598786							
Viperinae	*Atheris*	*ceratophora*		4	DQ305410	DQ305433		JF825388		DQ305474							
Viperinae	*Atheris*	*chlorechis*		9	EU624244	EU624278		AJ275679		EU624211	KX694697	KX694773	KX694904	KX694995		KX695062	
Viperinae	*Atheris*	*desaixi*		2		AJ275733		AJ275680									
Viperinae	*Atheris*	*hispida*		2		AJ275734		AJ275681									
Viperinae	*Atheris*	*mabuensis*	AT822	2				MF598824		MF598799							
Viperinae	*Atheris*	*matildae*	MTSN7570	2				JF825389		MF598801							
Viperinae	*Atheris*	*nitschei*		5	AY223650	AY223663		AF471070		AY223618		AF471125					
Viperinae	*Atheris*	*rungweensis*	SHCP07-R-02	2				MF598830		MF598808							
Viperinae	*Atheris*	*squamigera*		5	AF544762	EU624279		EU624303		EU624212		AF544734					
Viperinae	*Bitis*	*albanica*	WW1982	4		MK387628			MK387427						MK387568		MK387492
Viperinae	*Bitis*	*arietans*	1202	11	KX694571	MK387629	EU852310	JX114014	MK387428	JX114185			KX694906	KX694996	JX073299	KX695063	MK387493
Viperinae	*Bitis*	*armata*	1729	4		MK387636			JX073291						JX073302		MK387503
Viperinae	*Bitis*	*atropos*	1445	7	EU624246	EU624281		AJ275691	JX073287	EU624214					JX073298		MK387507
Viperinae	*Bitis*	*caudalis*	1752	7	EU624247	EU624282		AJ275693	JX073293	EU624215					JX073304		MK387513
Viperinae	*Bitis*	*cornuta*	1379	7	EU624248	EU624283		EU624305	MK387450	EU624216					MK387590		MK387520
Viperinae	*Bitis*	*gabonica*	1873	13	EU624249	EU624284	KX012772	AJ275695	JX073296	EU624217	KX694698	KX694822	KX694907	KX694997	JX073307	KX695064	MK387523
Viperinae	*Bitis*	*inornata*	Bit10, 13042	5		MK387660			MK387457	MK410937					MK387596		MK387528
Viperinae	*Bitis*	*nasicornis*	1241	9	DQ305411	AY188048	KX012786	AY188009	MK387458	DQ305475		AF471130			MK387597		MK387529
Viperinae	*Bitis*	*parviocula*	2980	4		MK387665			JX073292						JX073303		MK387533
Viperinae	*Bitis*	*peringueyi*	2817	7	DQ305412	DQ305435		DQ305458	MK387464	DQ305476					MK387603		MK387536
Viperinae	*Bitis*	*rhinoceros*	1287	7	EU624250	EU624285		AJ275696	MK387466	EU624218					MK387605		MK387538
Viperinae	*Bitis*	*rubida*	1397	7	EU624251	EU624286		EU624306	JX073290	EU624219					JX073301		MK387540
Viperinae	*Bitis*	*schneideri*	2811	4		MK387675			JX073297						JX073308		MK387545
Viperinae	*Bitis*	*worthingtoni*	1369	7	EU624252	AJ275745		AJ275692	JX073295	EU624220					JX073306		MK387548
Viperinae	*Bitis*	*xeropaga*	1556	7	EU624253	EU624287		EU624307	JX073294	EU624221					JX073305		MK387552
Viperinae	*Causus*	cf. *bilineatus*	Eli_216	2		MK410884		MK410887									
Viperinae	*Causus*	*defilippii*	MBUR_00840	11	GU045452	GU045452	GU045452	GU045452	GU045452	GU045452	EU402633	MK410890		EU390913	JN880816		JN614053
Viperinae	*Causus*	*lichtensteinii*	Eli_3099	8	KX694560	MK410885		MK410888			KX694705	MK410891	KX694916	KX695002		KX695072	
Viperinae	*Causus*	*maculatus*	Eli_2122	3		MK410886		MK410889				MK410892					
Viperinae	*Causus*	*resimus*		5	AY223649	AY223662		AY223555		AY223616		AF544696					
Viperinae	*Causus*	*rhombeatus*	WC_2691	5	DQ305409	DQ305432		DQ305455		U41866		MK410893					
Viperinae	*Cerastes*	*cerastes*		12	EU852317	EU852323	EU852311	AF471028		EU624222	KX694706	AF544679	KX694917	KX695003	HM623449	EU852329	HM623455
Viperinae	*Cerastes*	*gasperettii*		3	HQ658423	HQ267799		AJ275704									
Viperinae	*Cerastes*	*vipera*		2		AJ275757		AJ275705									
Viperinae	*Daboia*	*mauritanica*		4	EU624261	EU624295		KJ415304		EU624229							

					mtDNA						nDNA						
**Clade**	**Genus**	**Species**	**Sample**	**Genes**	**12S**	**16S**	**COI**	**CYTB**	**ND2**	**ND4**	**BDNF**	**CMOS**	**JUN**	**NT3**	**PRLR**	**RAG1**	**UBN1**
Viperinae	*Daboia*	*palaestinae*		3	JN870183	AJ275775		AJ275722									
Viperinae	*Daboia*	*russelii*		10	EU913478	EU913478	EU913478	EU913478	EU913478	EU913478		GQ225671		EU390916	JN880826		JN614055
Viperinae	*Daboia*	*siamensis*		5			AB920195	AB920249			EU402636	AF471156				EU402843	
Viperinae	*Echis*	*borkini*		4	GQ359644	GQ359734		GQ359485		GQ359569							
Viperinae	*Echis*	*carinatus*		8	EU852313	EU852319	EU852307	GQ359433		GQ359521					HM623447	EU852325	HM623453
Viperinae	*Echis*	*coloratus*		8	EU852315	HQ658452	EU852309	EU852297		EU852303					HM623444	EU852327	HM623450
Viperinae	*Echis*	*jogeri*		4	GQ359641	GQ359725		GQ359476		GQ359567							
Viperinae	*Echis*	*khosatzkii*		4	GQ359617	GQ359698		GQ359449		GQ359534							
Viperinae	*Echis*	*leucogaster*		4	GQ359621	GQ359704		GQ359455		GQ359540							
Viperinae	*Echis*	*ocellatus*		8	EU852312	GQ359731	EU852306	GQ359418		AF292607					HM623446	EU852324	HM623452
Viperinae	*Echis*	*omanensis*		4	GQ359631	GQ359723		GQ359466		GQ359550							
Viperinae	*Echis*	*pyramidum*		8	EU852314	EU852320	EU852308	EU852296		EU852302					HM623445	EU852326	HM623451
Viperinae	*Eristicophis*	*macmahoni*		4	EU624259	EU624293		HM179464		EU624227							
Viperinae	*Macrovipera*	*lebetina*		9	EU624260	EU624294		KJ415300		DQ897729	KX694736	KX694785	KX694942	KX695028		KX695096	
Viperinae	*Macrovipera*	*razii*		1				MF445994									
Viperinae	*Macrovipera*	*schweizeri*		3	EU624262	AJ275768		AJ275715									
Viperinae	*Montivipera*	*albizona*		6	EU624265	AJ275780	KX168823	KX168722		EU624231						KX169130	
Viperinae	*Montivipera*	*bornmuelleri*		4		AJ275779	KX168831	KX168730								KX169134	
Viperinae	*Montivipera*	*bulgardaghica*		4		AJ275783	KX168839	KX168738								KX169138	
Viperinae	*Montivipera*	*kuhrangica*		1			KJ950754										
Viperinae	*Montivipera*	*latifii*		5	JN870191	JN870199	KX168843	KX168742								KX169139	
Viperinae	*Montivipera*	*raddei*		9	KX694554	AJ275784	KX168856	KX168755			KX694739	KX694787	KX694945	KX695031		KX169144	
Viperinae	*Montivipera*	*wagneri*		6	JN870188	AJ275778	KX168872	KX168770		JN870213						KX169145	
Viperinae	*Montivipera*	*xanthina*		6	EU624268	AJ275777	KX168908	KX168806		EU624234						KX169155	
Viperinae	*Proatheris*	*superciliaris*		4	EU624263	EU624296		AJ275685		EU624230							
Viperinae	*Pseudocerastes*	*fieldi*		2	EU624264			AJ275716									
Viperinae	*Pseudocerastes*	*persicus*		2		AJ275769		AJ275717									
Viperinae	*Pseudocerastes*	*urarachnoides*		1				KF314714									
Viperinae	*Vipera*	*altaica*		2			KC122717	KC176729									
Viperinae	*Vipera*	*ammodytes*		7	L01768	EU624297	KU986335	DQ186508	AY321072	EU624232						KX357729	
Viperinae	*Vipera*	*anatolica*		2			KC122762	KC316113									
Viperinae	*Vipera*	*aspis*		6	JN870190		KP697881	KR153577	AY321085	JX649606						KX357730	
Viperinae	*Vipera*	*barani*		1				AY321092									
Viperinae	*Vipera*	*berus*		12	EU543221	KJ128962	KC122718	DQ186031	AY321075	FR727036	LT220962	KX694795	KX694975	LT220980	LT221007	KX357727	
Viperinae	*Vipera*	*dinniki*		3		AJ275773	KC122719	KC176731									
Viperinae	*Vipera*	*eriwanensis*		9	KX694555	KX694672	KC122722	FR727090		FR727021	KX694770	KX694794	KX694974	KX695056			
Viperinae	*Vipera*	*graeca*		5				LT220960		LN835177	LT220966			LT220984	LT221011		
Viperinae	*Vipera*	*kaznakovi*		3			KC122724	KC176736		FR727034							

Clade	Genus	Species	Sample	Genes	mtDNA						nDNA						
					**12S**	**16S**	**COI**	**CYTB**	**ND2**	**ND4**	**BDNF**	**CMOS**	**JUN**	**NT3**	**PRLR**	**RAG1**	**UBN1**
Viperinae	*Vipera*	*latastei*		3				AY321094	AY321074	JX649540							
Viperinae	*Vipera*	*lotievi*		3			KC122728	FR727097		FR727030							
Viperinae	*Vipera*	*monticola*		1				KJ415298									
Viperinae	*Vipera*	*orlovi*		2			KC122734	KC176746									
Viperinae	*Vipera*	*renardi*		6			KC122716	FR745991		FR727033	LT220977			LT220995	LT221022		
Viperinae	*Vipera*	*seoanei*		5		AJ275782	KC122736	DQ186030	AY321071	FR727035							
Viperinae	*Vipera*	*transcaucasiana*		2				DQ186478									
Viperinae	*Vipera*	*ursinii*		9	EF012817		KC122737	KC176749	AY321069	FR726956	LT220979	AF433658		LT220997	LT221009		
Viperinae	*Vipera*	*walser*		4		KX357758		KX357741		KX357731						KX357728	
**outgroup**																	
Azemiopinae	*Azemiops*	*feae*	*	10	AF512748	AF512748		KF997880		U41865	EU402628	KX694774	KX694905	KX694977		EU402836	JN614048
Crotalinae	*Agkistrodon*	*bilineatus*		4	AF156591	AF057277		EU483408		AF156583							
Crotalinae	*Agkistrodon*	*contortrix*	*	10	AF259224	AF259117	KU985795	EU483255		U41868	EU402623			JN703027	JN880801	EU402833	JN614044
Crotalinae	*Agkistrodon*	*howardgloydi*		3	AF156592	AF156571				AF156584							
Crotalinae	*Agkistrodon*	*piscivorus*		11	NC_009768	NC_009768	NC_009768	NC_009768	NC_009768	NC_009768	KX694693	KX694772	KX694900	KX694976		KX695059	
Crotalinae	*Agkistrodon*	*russeolus*		2		AF156573				AF156586							
Crotalinae	*Agkistrodon*	*taylori*		4	AF156589	AF156568		EU483477		AF156580							
Crotalinae	*Atropoides*	*indomitus*		1						KC354700							
Crotalinae	*Atropoides*	*mexicanus*		4	KC847268	KC847255		KC847271		KC847289							
Crotalinae	*Atropoides*	*nummifer*		5	DQ305422	AF057254		AY220314		AY220329						KU234642	
Crotalinae	*Atropoides*	*occiduus*		4	DQ305423	DQ305446		DQ061194		DQ061219							
Crotalinae	*Atropoides*	*olmec*		4	AY223656	AY223669		AY223585		AY223632							
Crotalinae	*Atropoides*	*picadoi*		4	AF057208	AF057255		AY223583		U41872							
Crotalinae	*Bothriechis*	*aurifer*		5	DQ305425	DQ305448		DQ305466		DQ305483						KU234643	
Crotalinae	*Bothriechis*	*bicolor*		5	DQ305426	DQ305449		DQ305467		DQ305484						KU234644	
Crotalinae	*Bothriechis*	*guifarroi*		5	KC847267	KC847262		KC847275		KC847286						KU234640	
Crotalinae	*Bothriechis*	*lateralis*		6	AF057211	AF057258	KU986328	AY223588		U41873						KU234645	
Crotalinae	*Bothriechis*	*marchi*		5	DQ305428	DQ305451		DQ305469		DQ305486						KU234646	
Crotalinae	*Bothriechis*	*nigroviridis*		5	AF057212	AF057259		AY223589		AY223635						KU234627	
Crotalinae	*Bothriechis*	*nubestris*		4		KU176473		KU203347		KU215602						KU234634	
Crotalinae	*Bothriechis*	*rowleyi*		5	DQ305427	DQ305450		DQ305468		DQ305485						KU234647	
Crotalinae	*Bothriechis*	*schlegelii*		7	AF057213	KC847256	KU986324	AY223590		U41874	FJ433983					KU234648	
Crotalinae	*Bothriechis*	*supraciliaris*		5	DQ305429	DQ305452		DQ305470		DQ305487						KU234649	
Crotalinae	*Bothriechis*	*thalassinus*		5	DQ305424	DQ305447		DQ305465		DQ305482						KU234650	
Crotalinae	*Bothrocophias*	*campbelli*		2				AF191582		AF292622							
Crotalinae	*Bothrocophias*	*hyoprora*		4	AF057206	AF057253		AY223593		U41886							
Crotalinae	*Bothrocophias*	*microphthalmus*		4	AY223657	AY223670		FR691565		FR691538							
Crotalinae	*Bothrops*	*alcatraz*		1				AY865820									
Crotalinae	*Bothrops*	*alternatus*		4	AY027777	AY223673		EU867273		AF292617							

Clade	Genus	Species	Sample	Genes	mtDNA						nDNA						
					**12S**	**16S**	**COI**	**CYTB**	**ND2**	**ND4**	**BDNF**	**CMOS**	**JUN**	**NT3**	**PRLR**	**RAG1**	**UBN1**
Crotalinae	*Bothrops*	*ammodytoides*		4	AY046572	AY223671		AY223595		AY223639							
Crotalinae	*Bothrops*	*asper*		9	AF057218	AF057265	KU986307	HE867043		U41876	EU402630			EU390910		EU402838	JN614050
Crotalinae	*Bothrops*	*atrox*		10	AY223659	AY223672	KU986342	GQ428476		AY223641	KX694699	KX694802	KX694908	KX694978		DQ469790	
Crotalinae	*Bothrops*	*barnetti*		1			KU986339										
Crotalinae	*Bothrops*	*bilineatus*		4	AF057214	AF057261		AY223591		U41875							
Crotalinae	*Bothrops*	*brazili*		7	EU867252	EU867264	KU986320	EU867276		AF292635		KX694775				KX695066	
Crotalinae	*Bothrops*	*caribbaeus*		2				AF292598		AF292636							
Crotalinae	*Bothrops*	*chloromelas*		4	DQ305430	DQ305453		DQ305471		DQ305488							
Crotalinae	*Bothrops*	*cotiara*		4	AF057217	AF057264		AY223597		AF292619							
Crotalinae	*Bothrops*	*diporus*		4	DQ305431	DQ305454		KF801111		AF292625							
Crotalinae	*Bothrops*	*erythromelas*		4	AF057219	AF057266		AY223600		KF801255							
Crotalinae	*Bothrops*	*fonsecai*		7	KX694552	KX694621		AF292580		AF292618		KX694803	KX694909			KX695067	
Crotalinae	*Bothrops*	*insularis*		4	AF057216	AF057263		AY223596		AF188705							
Crotalinae	*Bothrops*	*itapetiningae*		4	EU867253	EU867265		AF292582		AF292620							
Crotalinae	*Bothrops*	*jararaca*		5	EU867254	EU867266		AF292589		AF292627				KX694979			
Crotalinae	*Bothrops*	*jararacussu*		8	AY223661	AY223674		AY223602		AF292634		KX694776	KX694910	KX694980		KX695068	
Crotalinae	*Bothrops*	*lanceolatus*		2				AF292599		AF292637							
Crotalinae	*Bothrops*	*leucurus*		5	EU867255	EU867267	KU986310	EU867279		AF246279							
Crotalinae	*Bothrops*	*lojanus*		2				FR691566		FR691536							
Crotalinae	*Bothrops*	*lutzi*		2				KF801130		KF801261							
Crotalinae	*Bothrops*	*marajoensis*		2				AF292605		AF292643							
Crotalinae	*Bothrops*	*marmoratus*		2				KF801137		KF801265							
Crotalinae	*Bothrops*	*matogrossensis*		2				KF801148		KF801276							
Crotalinae	*Bothrops*	*moojeni*		4	EU867256	EU867268		AF200222		AF292644							
Crotalinae	*Bothrops*	*neuwiedi*		4		JQ627282	JQ627371	AF292585		KF801287							
Crotalinae	*Bothrops*	*oligolepis*		3		KX660260				KX660646				KX652071			
Crotalinae	*Bothrops*	*osbornei*		4	KU999231	KU999161		KU999114		AF292633							
Crotalinae	*Bothrops*	*pauloensis*		4	EU867260	EU867272		EU867284		EU867296							
Crotalinae	*Bothrops*	*pictus*		2				AF292583		AF292621							
Crotalinae	*Bothrops*	*pubescens*		4	JN870180	JN870192		JN870200		KF801343							
Crotalinae	*Bothrops*	*pulchra*		3	JN870179			AF292593		AF292631							
Crotalinae	*Bothrops*	*punctatus*		4	KU999235	KU999166		KU999118		AF292632							
Crotalinae	*Bothrops*	*taeniatus*		4	AF057215	AF057262		AY223592		AF292629							
Crotalinae	*Calloselasma*	*rhodostoma*		9	AF057190	AF057237		AY223562		U41878	KX694703	KX694777	KX694914	KX694981		KX695070	
Crotalinae	*Cerrophidion*	*godmani*		5	DQ305419	AF057250		HE867046		U41879						KU234653	
Crotalinae	*Cerrophidion*	*petlalcalensis*		4	DQ305420	DQ305443		DQ061202		DQ061227							
Crotalinae	*Cerrophidion*	*tzotzilorum*		4	JN870182	JN870193		DQ061203		DQ061228							
Crotalinae	*Cerrophidion*	*wilsoni*		4	JQ724143	JQ627129		JQ724156		JQ724175							
Crotalinae	*Crotalus*	*adamanteus*		9	AF259255	AF259147	KU985639	KJ730274		JN620959		KX694780	KX694921	KX694982		JN621000	
Crotalinae	*Crotalus*	*aquilus*		4	AF259232	AF259125		AF259162		HQ257762							

					mtDNA						nDNA						
**Clade**	**Genus**	**Species**	**Sample**	**Genes**	**12S**	**16S**	**COI**	**CYTB**	**ND2**	**ND4**	**BDNF**	**CMOS**	**JUN**	**NT3**	**PRLR**	**RAG1**	**UBN1**
Crotalinae	*Crotalus*	*armstrongi*		3	HQ257566	HQ257529				HQ257775							
Crotalinae	*Crotalus*	*atrox*		9	AF259256	AF259148	KU985749	JN620810	AY016239	AY223646		JN620892		KX256628		JN621001	
Crotalinae	*Crotalus*	*basiliscus*		6	AF259244	AF259136		AY704844	AY704796	AY704894				KF410313			
Crotalinae	*Crotalus*	*catalinensis*		3	AF259259	AF259151		AF259189									
Crotalinae	*Crotalus*	*cerastes*		9	AF259235	AF259128	KU985599	U69773	AY016245	JN620962		JN620894		KF410314		JN621003	
Crotalinae	*Crotalus*	*cerberus*		4				JN620813	AY016225	JN620963		JN620895					
Crotalinae	*Crotalus*	*culminatus*		3				AF393627	AY704786	AY704877							
Crotalinae	*Crotalus*	*durissus*		6	AF259248	AF259138	JQ627373	AY196645	AY704784	AF194168							
Crotalinae	*Crotalus*	*enyo*		4	AF259245	AF259137		AF259175	AY016246								
Crotalinae	*Crotalus*	*ericsmithi*		5				KF410284		KF410289		KF410301		KF410315		KF410330	
Crotalinae	*Crotalus*	*horridus*		9	NC_014400	NC_014400	NC_014400	NC_014400	NC_014400	NC_014400		JN620897		KF410316		JN621004	
Crotalinae	*Crotalus*	*intermedius*		3	AF259238	AF259131				JN870208							
Crotalinae	*Crotalus*	*lannomi*		5				KF410280		KF410291		KF410303		KF410317		KF410331	
Crotalinae	*Crotalus*	*lepidus*		7	AF259230	AF259123	KU985625	JN620816		U41881		JN620898				JN621005	
Crotalinae	*Crotalus*	*mitchellii*		8	AF259250	AF259142	KU985590	JN620817	AY016241	JN620967		JN620899				JN621006	
Crotalinae	*Crotalus*	*molossus*		8	AF259243	AF259135	KU985681	JN620822	AY704797	AY223645		JN620902				JN621009	
Crotalinae	*Crotalus*	*oreganus*		6	DQ020024		KU985566	JN620814	AY016217	U41882		AF471135					
Crotalinae	*Crotalus*	*polystictus*		7	AF259236	AF259129		KF410281		KF410294		KF410305		KF410320		KF410332	
Crotalinae	*Crotalus*	*pricei*		7	AF259237	AF259130	KU985692	AF259167		JN022844		KF410306		KF410321			
Crotalinae	*Crotalus*	*pusillus*		4	AF259229	AF259122		AF259159		HQ257774							
Crotalinae	*Crotalus*	*ravus*		4	AF259228	AF259121		AY223609		AY223647							
Crotalinae	*Crotalus*	*ruber*		6	AF259261	AF259152	KU985635	KP765661		DQ679838		KP765643					
Crotalinae	*Crotalus*	*scutulatus*		7	AF259254	AF259146	KU985747	AF292571	AY016237	AF292609				KX256582			
Crotalinae	*Crotalus*	*simus*		3	EU624240	GQ372869		HE867034									
Crotalinae	*Crotalus*	*stejnegeri*		5				KF410283		KF410295		KF410307		KF410323		KF410333	
Crotalinae	*Crotalus*	*tancitarensis*		3	JN022897	JN022897				JN022842							
Crotalinae	*Crotalus*	*tigris*	*	9	AF259249	AF259141	KU985565	JN620818	AY016240	AF156574		JN620900		GQ334665		JN621007	
Crotalinae	*Crotalus*	*totonacus*		3				AY704837	AY704795	AY704887							
Crotalinae	*Crotalus*	*transversus*		4	JN022895	JN022895		AF259169		JN022840							
Crotalinae	*Crotalus*	*triseriatus*		6	AF259233	AF259124		AF259163		HQ257761		KF410310		KF410325			
Crotalinae	*Crotalus*	*tzabcan*		3				AF393625	AY704791	AY704856							
Crotalinae	*Crotalus*	*viridis*		9	DQ020027	AF259145	KU985701	JN620819	AY016218	JN620969		JN620901		KX256604		JN621008	
Crotalinae	*Crotalus*	*willardi*		9	AF259240	AF259132	KU985952	AF259170		JN870209	KX694712	KX694781		KF410326		KX695078	
Crotalinae	*Deinagkistrodon*	*acutus*		11	NC_010223	NC_010223	NC_010223	NC_010223	NC_010223	NC_010223	KX694713	KX694782	KX694923	KX694984		KX695079	
Crotalinae	*Garthius*	*chaseni*		4	AY352791	AY352729		AY352760		AY352825							
Crotalinae	*Gloydius*	*blomhoffii*		3	EF012800			JF357942		U41867							
Crotalinae	*Gloydius*	*brevicaudus*		7	NC_011390	NC_011390	NC_011390	NC_011390	NC_011390	NC_011390		JF827697					
Crotalinae	*Gloydius*	*halys*	*	9	AF057191	AF057238	KR046004	AY223564	AY662540	JQ356856	KX694722			KX694985		AY662614	
Crotalinae	*Gloydius*	*intermedius*		7	NC_025560	NC_025560	NC_025560	NC_025560	NC_025560	NC_025560		JQ687507					
Crotalinae	*Gloydius*	*lijianlii*		2				KF997904		KF997956							

					mtDNA						nDNA						
**Clade**	**Genus**	**Species**	**Sample**	**Genes**	**12S**	**16S**	**COI**	**CYTB**	**ND2**	**ND4**	**BDNF**	**CMOS**	**JUN**	**NT3**	**PRLR**	**RAG1**	**UBN1**
Crotalinae	*Gloydius*	*liupanensis*		3				JQ687491		JQ687472		JQ687510					
Crotalinae	*Gloydius*	*monticola*		2				KF997903		KF997977							
Crotalinae	*Gloydius*	*rickmersi*		1						KM078592							
Crotalinae	*Gloydius*	*saxatilis*		7	NC_025666	NC_025666	NC_025666	NC_025666	NC_025666	NC_025666		JQ687508					
Crotalinae	*Gloydius*	*shedaoensis*		7	NC_029424	NC_029424	NC_029424	NC_029424	NC_029424	NC_029424		JQ687517					
Crotalinae	*Gloydius*	*strauchi*		9	EF012814	AF057239		JQ687490		JQ687471	KX694723	KX694784	KX694932	KX694986		KX695088	
Crotalinae	*Gloydius*	*tsushimaensis*		4	JN870186	JN870196		JN870203		JN870211							
Crotalinae	*Gloydius*	*ussuriensis*		7	NC_026553	NC_026553	NC_026553	NC_026553	NC_026553	NC_026553		JQ687520					
Crotalinae	*Hypnale*	*hypnale*		4	AF057189	AF057236		AY352750		U41884							
Crotalinae	*Hypnale*	*nepa*		6	KC347325	KC347362		KC347485		KC347491		KC347401				KC347439	
Crotalinae	*Hypnale*	*zara*		6	KC347326	KC347363		KC347463		KC347513		KC347402				KC347440	
Crotalinae	*Lachesis*	*acrochorda*		4	JN870187	JN870197		JN870204		JN870212							
Crotalinae	*Lachesis*	*melanocephala*		2				U96018		U96028							
Crotalinae	*Lachesis*	*muta*		6	AF057221	AF057268		AY223604		AY223644				EU390924	JN880845		
Crotalinae	*Lachesis*	*stenophrys*		5	AF057220	AF057267		AY223603		U96026	EU402644						
Crotalinae	*Mixcoatlus*	*barbouri*		4	HM363639	HM363640		HM363641		HM363642							
Crotalinae	*Mixcoatlus*	*browni*		4	HM363643	HM363644		HM363649		HM363653							
Crotalinae	*Mixcoatlus*	*melanurus*		4	AF057210	AF057257		AY223587		AY223634							
Crotalinae	*Ophryacus*	*undulatus*		4	AF057209	AF057256		AY223586		AY223633							
Crotalinae	*Ovophis*	*convictus*		4	HQ325265	HQ325083		HQ325141		HQ325190							
Crotalinae	*Ovophis*	*monticola*		10	AY763192	AY763211		HQ325124		AY059582	KX694744	KX694788		KX694987	KT220344	KX695103	KT220381
Crotalinae	*Ovophis*	*okinavensis*		6	AB175670	AB175670	AB175670	AB175670	AB175670	AB175670							
Crotalinae	*Ovophis*	*tonkinensis*		4	HQ325308	HQ325092		HQ325132		HQ325191							
Crotalinae	*Ovophis*	*zayuensis*		4	HQ325294	HQ325118		HQ325150		HQ325208							
Crotalinae	*Porthidium*	*arcosae*		5	EU624241	GQ372871		AF292575		AF292613						KU234654	
Crotalinae	*Porthidium*	*dunni*		4	AY223654	AY223667		AY223581		AY223630							
Crotalinae	*Porthidium*	*hespere*		2				EU017535		EU016098							
Crotalinae	*Porthidium*	*lansbergii*		4	AY223655	GQ372870		DQ061205		DQ061230							
Crotalinae	*Porthidium*	*nasutum*		9	AF057204	AF057251		AY223579		DQ061232	KX694748	KX694790	KX694954	KX694988		KU234655	
Crotalinae	*Porthidium*	*ophryomegas*		4	AF057205	AF057252		AY223580		U41888							
Crotalinae	*Porthidium*	*porrasi*		4	DQ305421	DQ305444		DQ061211		DQ061236							
Crotalinae	*Porthidium*	*yucatanicum*		4	JN870189	JN870198		DQ061215		DQ061244							
Crotalinae	*Protobothrops*	*cornutus*		10	NC_022695	NC_022695	NC_022695	NC_022695	NC_022695	NC_022695		KT220399			KT220346	KT220363	KT220383
Crotalinae	*Protobothrops*	*dabieshanensis*		10	NC_022473	NC_022473	NC_022473	NC_022473	NC_022473	NC_022473		KT220400			KT220347	KT220364	KT220384
Crotalinae	*Protobothrops*	*elegans*		6	LC073748	LC073748	LC073748	LC073748	LC073748	LC073748							
Crotalinae	*Protobothrops*	*flavoviridis*		6	LC073706	LC073706	LC073706	LC073706	LC073706	LC073706							
Crotalinae	*Protobothrops*	*himalayanus*		10	NC_029165	NC_029165	NC_029165	NC_029165	NC_029165	NC_029165		KT220401			KT220348	KT220366	KT220385
Crotalinae	*Protobothrops*	*jerdonii*		7	NC_021402	NC_021402	NC_021402	NC_021402	NC_021402	NC_021402		KF997929					
Crotalinae	*Protobothrops*	*kaulbacki*		10	NC_029166	NC_029166	NC_029166	NC_029166	NC_029166	NC_029166		KT220402			KT220349	KT220367	KT220387
Crotalinae	*Protobothrops*	*mangshanensis*		10	NC_026052	NC_026052	NC_026052	NC_026052	NC_026052	NC_026052		JQ687524			KT220351	KT220368	KT220388

Clade	Genus	Species	Sample	Genes	mtDNA						nDNA						
					**12S**	**16S**	**COI**	**CYTB**	**ND2**	**ND4**	**BDNF**	**CMOS**	**JUN**	**NT3**	**PRLR**	**RAG1**	**UBN1**
Crotalinae	*Protobothrops*	*maolanensis*		10	NC_026051	NC_026051	NC_026051	NC_026051	NC_026051	NC_026051		KT220406			KT220352	KT220370	KT220389
Crotalinae	*Protobothrops*	*mucrosquamatus*	*	12	NC_021412	NC_021412	NC_021412	NC_021412	NC_021412	NC_021412	XM_015815390	KT220410	XM_015831561		KT220356	XM_015822485	KT220393
Crotalinae	*Protobothrops*	*sieversorum*		8	KT220280	DQ305437		KT220320		DQ305478		KT220414			KT220360	KT220378	KT220395
Crotalinae	*Protobothrops*	*tokarensis*		6	NC_030182	NC_030182	NC_030182	NC_030182	NC_030182	NC_030182							
Crotalinae	*Protobothrops*	*trungkhanhensis*		8	KT220282	KT220302		KT220321		KT220341		KT220417			KT220361	KT220379	KT220397
Crotalinae	*Protobothrops*	*xiangchengensis*		7	KF460436	KF460436	KF460436	KF460436	KF460436	KF460436		KF997928					
Crotalinae	*Sistrurus*	*catenatus*	*	8	AF259226	AF259119	KU986216	AY223610	JQ609661	AF156575		KF410311		GQ334686			
Crotalinae	*Sistrurus*	*miliarius*		6	AF259227	AF259120	KU985759	EU483385	GQ359815	HQ257760							
Crotalinae	*Trimeresurus*	*albolabris*		8	NC_022820	NC_022820	NC_022820	NC_022820	NC_022820	NC_022820				KP999637		KP999549	
Crotalinae	*Trimeresurus*	*andersonii*		3	AY352801	AY352740				AY352835							
Crotalinae	*Trimeresurus*	*borneensis*		4	AY352783	AY352722		AY352754		AY352817							
Crotalinae	*Trimeresurus*	*buniana*		6		KX660233		KX660502		KX660631		KX660374		KX652049		KX660131	
Crotalinae	*Trimeresurus*	*cantori*		4	AF057196	AF057243		AY223568		U41891							
Crotalinae	*Trimeresurus*	*cardamomensis*		6	KR021095	KR021129				KR021061		KX660361		KX652036		KX660120	
Crotalinae	*Trimeresurus*	*erythrurus*		4	AF517161	AF517174		AY352768		AF517217							
Crotalinae	*Trimeresurus*	*fasciatus*		4	GQ428492	GQ428466		GQ428475		GQ428482							
Crotalinae	*Trimeresurus*	*flavomaculatus*		4	AY059535	AY352734		AF171916		AY352830							
Crotalinae	*Trimeresurus*	*fucatus*		6		KX660235		KX660505		KX660633		KX660376		KX652051		KX660133	
Crotalinae	*Trimeresurus*	*gracilis*		4	DQ305415	DQ305438		DQ305461		DQ305479							
Crotalinae	*Trimeresurus*	*gramineus*		4	AY352793	AY352731		AY352762		AY352828							
Crotalinae	*Trimeresurus*	*gumprechti*		6	KT216287	GQ428461	KC171170	KT216377		AY352832				KX019436			
Crotalinae	*Trimeresurus*	*hageni*		4	AY371755	GQ428462		AY059567		AY371867							
Crotalinae	*Trimeresurus*	*honsonensis*		5		KX660222				KX660620		KX660363		KX652038		KX660121	
Crotalinae	*Trimeresurus*	*insularis*		4	AF517159	AF517172		AY059568		AF517215							
Crotalinae	*Trimeresurus*	*kanburiensis*		4	AY289219	AY352737		AY289225		AY289231							
Crotalinae	*Trimeresurus*	*macrops*		5	AF517163	AF517176		KP999371		KP999465				KP999625			
Crotalinae	*Trimeresurus*	*malabaricus*		4	AY059548	AY352733		AY352763		AY352829							
Crotalinae	*Trimeresurus*	*malcolmi*		4	AY371758	AY371793		AY371832		AY371861							
Crotalinae	*Trimeresurus*	*mcgregori*		4	AY371756	AY371795		AY371831		AY371858							
Crotalinae	*Trimeresurus*	*medoensis*		4	AY352797	AY352735		AY352765		AY352831							
Crotalinae	*Trimeresurus*	*nebularis*		6		KX660236		KX660506		KX660634		KX660377		KX652052		KX660134	
Crotalinae	*Trimeresurus*	*popeiorum*		4	KT216266	AY371768		KT216359		AY371835							
Crotalinae	*Trimeresurus*	*puniceus*		4	AF517164	AF517177		AY352757		AY352820							
Crotalinae	*Trimeresurus*	*purpureomaculatus*		4	AY352806	AF517175		AY352772		AY352841							
Crotalinae	*Trimeresurus*	*rubeus*		3	KR021105	KR021141				KR021063							
Crotalinae	*Trimeresurus*	*sabahi*		2				KP899261		KP939319							
Crotalinae	*Trimeresurus*	*schultzei*		4	AY352785	AY352725		AY352756		AY352819							
Crotalinae	*Trimeresurus*	*septentrionalis*		6	AY352784	AY352724		KP999348		KP999443				KP999618		KP999536	
Crotalinae	*Trimeresurus*	*sichuanensis*		6	NC_029494	NC_029494	NC_029494	NC_029494	NC_029494	NC_029494							
Crotalinae	*Trimeresurus*	*stejnegeri*		11	NC_012146	NC_012146	NC_012146	NC_012146	NC_012146	NC_012146	KX694769	KX694793	KX694973	KX019417		KX695119	

					mtDNA						nDNA						
**Clade**	**Genus**	**Species**	**Sample**	**Genes**	**12S**	**16S**	**COI**	**CYTB**	**ND2**	**ND4**	**BDNF**	**CMOS**	**JUN**	**NT3**	**PRLR**	**RAG1**	**UBN1**
Crotalinae	*Trimeresurus*	*sumatranus*		4	AY371759	AY371788		AY371824		AY371865							
Crotalinae	*Trimeresurus*	*tibetanus*		4	AY352776	AY352715		AY352749		AY352810							
Crotalinae	*Trimeresurus*	*trigonocephalus*		6	KC347336	AY059565	KU986306	KC347479		AY059597		KC347412					
Crotalinae	*Trimeresurus*	*truongsonensis*		4	EU443817	EU443818		EU443815		EU443816							
Crotalinae	*Trimeresurus*	*venustus*		4	AY293931	AY352723		AF171914		KR021049							
Crotalinae	*Trimeresurus*	*vogeli*		5	AF517170	AF517183		AY059574		AF517225				KX019315			
Crotalinae	*Trimeresurus*	*wiroti*		1				DQ646788									
Crotalinae	*Trimeresurus*	*yunnanensis*		6	EU443811	EU443812	KC171173	EF597523		EF597528				KX019380			
Crotalinae	*Tropidolaemus*	*subannulatus*		4		KX660255		KX660525		KX660644		KX660396					
Crotalinae	*Tropidolaemus*	*wagleri*		5	AF057198	AF057245	KU986318	GQ428472		U41896							
Boidae	*Boa*	*constrictor*	*	12	NC_007398	NC_007398	NC_007398	NC_007398	NC_007398	NC_007398	AY988030	AF544676		HQ399534	JN880812	KF576890	JF806219
Colubridae	*Coronella*	*austriaca*	*	10	KX694589	KX694634	KC997577	KX694868	AY487026	AY487065	KX694711	AY486954		KX695008		KX695077	
Colubridae	*Eirenis*	*modestus*	*	10	AY039143	KX694641	AY039181	KX694864	AY487033	AY487072	KX694718	KX694807		KX695014		KX695084	
Colubridae	*Gonyosoma*	*oxycephalum*	*	11	AY122678	KX694646	LC075339	KX694870	DQ902241	DQ902309	KX694725	KX694808	KX694934	KX695018		KX695090	
Colubridae	*Pituophis*	*deppei*	*	11	KX694582	KX694657	AY122741	KX694867	FJ627848	AF141096	KX694747	KX694814	KX694953	KX695037		KX695104	
Colubridae	*Spalerosophis*	*diadema*	*	11	KX694605	KX694668	AY039186	KX694865	AY487020	AY487059	KX694764	KX694820	KX694970	KX695051		KX695115	
Cylindrophiidae	*Cylindrophis*	*ruffus*	*	11	NC_007401	NC_007401	NC_007401	NC_007401	NC_007401	NC_007401	EU402635	AF471133	KX694922	AY988054		AY662613	
Elapidae	*Bungarus*	*fasciatus*	*	11	NC_011393	NC_011393	NC_011393	EU579523	NC_011393	EU579523	KX694700	KX694826	KX694911	KX694998		AY487389	
Elapidae	*Ophiophagus*	*hannah*	*	11	NC_011394	NC_011394	NC_011394	NC_011394	NC_011394	NC_011394	KX694743	KX694798	KX694950	KX695035		KX695102	

Supplementary Table S2. Genetic markers used for the phylogenetic analyses, their respective alignment lengths and rates of molecular evolution as resulting from the FBD analysis.

Genome	Gene	Source of primers and PCR protocols for newly generated sequences	Alignment length (bp)	Evolutionary rate (mean ± standard error)
mtDNA	12S		371	0.00423 ± 0.00001
	16S	Wüster et al. (2008)	429	0.00324 ± 0.00001
	cox1		944	0.01000 ± 0.00004
	cytb	Burbrink et al. (2000)	1131	0.01090 ± 0.00003
	nd2	Barlow et al. (2012)	1032	0.00976 ± 0.00003
	nd4	Wüster et al. (2008)	825	0.00706 ± 0.00002
nDNA	bdnf		668	0.00019 ± 5.32E-07
	cmos	Lawson et al. (2005)	719	0.00051 ± 1.39E-06
	jun		343	0.00046 ± 2.14E-06
	nt3		519	0.00117 ± 3.46E-06
	prlr	Barlow et al. (2012)	559	0.00096 ± 2.85E-06
	rag1		2405	0.00038 ± 1.14E-06
	ubn1	Barlow et al. (in press)	473	0.00094 ± 2.66E-06

Supplementary Table S3. A list of viper fossils that were used for calibrating divergence times under the FBD model, their phylogenetic position, minimum and maximum estimated stratigraphic age, and locality details. The ‘Notes’ field contains details regarding the morphology or phylogenetic affinity of some of the specimens as described in the reference cited.

Fossil	Phylogenetic affinity	Min age	Max age	Country	Locality	Lat	Long	Notes	Reference
Bitis arietans	Bitis arietans	2.5	2.5	Morocco	Ahl al Oughlam (near Casablanca)	33.5931	-7.6164		Geraads 2006
Bitis cf. olduvaiensis	sister to Bitis arietans	2.66	3.85	Tanzania	Laetoli	-2.9962	35.3524		Rage 1973
Bitis olduvaiensis	sister to Bitis arietans	1.7	1.845	Tanzania	Olduvai Rift Valley	-2.983	35.35	Fossil assignment unclear, can be either B. olduvaiensis or B. arietans	Rage 1973
Bitis sp.	Bitis	17	17.5	Namibia	Arrisdrift	-28.55	16.5	The earliest fossil record of the genus Bitis	Rage 2003
Daboia mauritanica	Daboia mauritanica	0.005	0.005	Morocco	El Harhoura 2 (Temara)	33.9522	-6.9259		Böhme and Ilg 2003
Daboia mauritanica	Daboia mauritanica	0.01	0.2	Morocco	El Harhoura 1 (Temara)	33.95	-6.9333		Böhme and Ilg 2003
Daboia maxima	Daboia	3.5	3.6	Spain	Layna, Province de Soria	41.1	-2.3		Böhme and Ilg 2003
Macrovipera burgenlandica	sister to M. gedulyi	8.55	8.95	Austria	Kohfidisch	47.1667	16.35	Typical member of the 'Oriental vipers'; display features characteristic for the living species M. lebetina, D. palaestinae, and M. xanthina, but not D. russelli	Bachmayer and Syzndlar 1987
Macrovipera gedulyi	sister to M. burgenlandica	5.35	6.15	Hungary	Polgardi	47.05	18.3	The prefrontal somewhat resembles that of M. lebetina, closely related with the extinct M. burgenlandica	Venczel 1998

Fossil	Phylogenetic affinity	Min age	Max age	Country	Locality	Lat	Long	Notes	Reference
Macrovipera gedulyi	sister to M. burgenlandica	5.33	6.2	Hungary	Polgardi	47.05	18.3	The prefrontal somewhat resembles that of M. lebetina, closely related with the extinct M. burgenlandica	Venczel 1998
Macrovipera sp.	Macrovipera	11.85	12.05	Romania	Taut, Arad district	46.2833	21.9333		Venczel and Stiuca 2008
Macrovipera kuchurganica	Macrovipera	4.85	5.25	Ukraine	Kuchurgan	46.75	29.9833	Considered a close relative of the extant Vipera lebetina	Szyndlar and Rage 2002
Montivipera xanthina	Montivipera xanthina	4.631	4.799	Armenia	Nurnus, Kotayk Province	40.3534	44.6082		Böhme and Ilg 2003
Vipera platyspondyla	A clade of Macrovipera + Montivipera	17	17.5	Czech Republic	Dolnice near Cheb	50.0833	12.3833	Appears closest to M. xanthina, but M. lebetina is morphologically variable in the characters examined so close relationship to this species cannot be ruled out	Szyndlar 1987
Vipera platyspondyla	A clade of Macrovipera + Montivipera	17.5	18	Germany	Petersbuch 2	48.9779	11.1909	Appears closest to M. xanthina, but M. lebetina is morphologically variable in the characters examined so close relationship to this species cannot be ruled out	Szyndlar 1987
Vipera sp.	A clade of Macrovipera + Montivipera	5	5.33	Spain	Calo d´en Rafelino, Manacor, Mallorca	39.5566	3.3647	The possible presence of a neural spine longer than high in the fossil trunk vertebra allows us to identify the fossil as belonging to the 'Oriental vipers' and differentiate it from Daboia, which displays a higher neural spine.	Bailon et al. 2010
'Oriental viper'	A likely non-monophyletic group of 'Oriental vipers'; genera Daboia, Montivipera, Macrovipera	2.5	2.5	Morocco	Ahl al Oughlam (near Casablanca)	33.5931	-7.6164		Böhme and Ilg 2003
'Oriental viper'	A likely non-monophyletic group of 'Oriental vipers'; genera Daboia, Montivipera, Macrovipera	6.8	7.7	Hungary	Tardosbanya 3	47.6667	18.45		Venczel 1998
'Oriental viper'	A likely non-monophyletic group of 'Oriental vipers'; genera Daboia, Montivipera, Macrovipera	8.1	8.8	Ukraine	Novoelizavetovka 2+3, lower layer	47.15	30.4055		Zerova 1993

Fossil	Phylogenetic affinity	Min age	Max age	Country	Locality	Lat	Long	Notes	Reference
‘Oriental viper’	A likely non-monophyletic group of 'Oriental vipers'; genera Daboia, Montivipera, Macrovipera	11	12	Russia	Togay, Olkhon Island, Baykal Lake	53.14	107.36	The marked shortness of the vertebra, the strongly depressed condition of the neural arch, and the relatively large size point to the ‘Oriental complex’	Rage and Danilov 2008
‘Oriental viper’	A likely non-monophyletic group of 'Oriental vipers'; genera Daboia, Montivipera, Macrovipera	12	13	France	Isle d’Abeau	45.6199	5.2243		Szyndlar and Rage 2002
‘Oriental viper’	A likely non-monophyletic group of 'Oriental vipers'; genera Daboia, Montivipera, Macrovipera	13.7	14.6	Germany	Petersbuch 39	48.9779	11.1909		Szyndlar and Rage 2002
‘Oriental viper’	A likely non-monophyletic group of 'Oriental vipers'; genera Daboia, Montivipera, Macrovipera	13.5	14.5	Russia	Belomechetskaya	44.4	41.9333		Szyndlar and Rage 2002
‘Oriental viper’	A likely non-monophyletic group of 'Oriental vipers'; genera Daboia, Montivipera, Macrovipera	14.8	14.8	Germany	Griesbeckerzell 1a	48.4468	11.0543		Ivanov and Bohme 2011
‘Oriental viper’	A likely non-monophyletic group of 'Oriental vipers'; genera Daboia, Montivipera, Macrovipera	14.9	14.9	Germany	Edelbeuren-Maurerkopf	48.0962	10.0311		Szyndlar and Rage 2002
‘Oriental viper’	A likely non-monophyletic group of 'Oriental vipers'; genera Daboia, Montivipera, Macrovipera	14	17.5	France	Vieux Collonges	45.75	4.85		Ivanov 2000
‘Oriental viper’	A likely non-monophyletic group of 'Oriental vipers'; genera Daboia, Montivipera, Macrovipera	15	15	Germany	Griesbeckerzell 1b	48.4468	11.0543		Szyndlar and Rage 2002
‘Oriental viper’	A likely non-monophyletic group of 'Oriental vipers'; genera Daboia, Montivipera, Macrovipera	16.27	16.47	Germany	Sandelzhausen	48.6283	11.796		Szyndlar 2009
‘Oriental viper’	A likely non-monophyletic group of 'Oriental vipers'; genera Daboia, Montivipera, Macrovipera	16.5	16.5	Germany	Schiessen	48.2974	10.2432		Szyndlar and Rage 2002

**Fossil**	**Phylogenetic affinity**	**Min age**	**Max age**	**Country**	**Locality**	**Lat**	**Long**	**Notes**	**Reference**
'Oriental viper'	A likely non-monophyletic group of 'Oriental vipers'; genera Daboia, Montivipera, Macrovipera	16.6	17.2	Portugal	Quinta das Pedreiras near Lumiar Lisboa	38.7167	-9.1333		Szyndlar 2000
'Oriental viper'	A likely non-monophyletic group of 'Oriental vipers'; genera Daboia, Montivipera, Macrovipera	17	17.5	France	Montréal-du-Gers	43.9542	0.1971		Szyndlar and Rage 2002
'Oriental viper'	A likely non-monophyletic group of 'Oriental vipers'; genera Daboia, Montivipera, Macrovipera	4.85	5.25	Ukraine	Kuchurgan	46.75	29.9833		Szyndlar and Rage 2002
'Oriental viper'	A likely non-monophyletic group of 'Oriental vipers'; genera Daboia, Montivipera, Macrovipera	5.25	5.5	Greece	Maramena 1	41.1791	23.4711		Szyndlar and Rage 2002
'Oriental viper'	A likely non-monophyletic group of 'Oriental vipers'; genera Daboia, Montivipera, Macrovipera	5.33	6	Italy	Brisighella Cava Monticino	44.2167	11.7667		Rook et al. 2015
'Oriental viper'	A likely non-monophyletic group of 'Oriental vipers'; genera Daboia, Montivipera, Macrovipera	7.6	8.7	Ukraine	Frunzovka 2	47.333	29.75		Szyndlar 1991
'Oriental viper'	A likely non-monophyletic group of 'Oriental vipers'; genera Daboia, Montivipera, Macrovipera	8	9	Georgia	Rustavi	41.5261	45.0353		Szyndlar and Rage 2002
Vipera ukrainica	A likely non-monophyletic group of 'Oriental vipers'; genera Daboia, Montivipera, Macrovipera	11.04	12.014	Ukraine	Gritsev (Khmelnitsk area, Shepetovski district)	49.975	27.16		Szyndlar and Rage 2002
Vipera sarmatica	A likely non-monophyletic group of 'Oriental vipers'; genera Daboia, Montivipera, Macrovipera	9.5	9.9	Moldova	Kalfa	46.9042	29.3753		Szyndlar 1991<br>Szyndlar and Rage 2002
Vipera ammodytes	V. ammodytes	0.005	0.127	Serbia	Smolucka Cave, near Novi Pazar	43.0517	20.3681		Böhme and Ilg 2003

Fossil	Phylogenetic affinity	Min age	Max age	Country	Locality	Lat	Long	Notes	Reference
Vipera ammodytes	V. ammodytes	0.127	0.7	Greece	Tourkobounia 2	37.99	23.49		Böhme and Ilg 2003
Vipera ammodytes	V. ammodytes	0.7	1.64	Czech Republic	Stranska Skala Hill near Brno	49.2	16.6333		Böhme and Ilg 2003
Vipera ammodytes	V. ammodytes	0.78	1.8	Austria	Bad Deutsch-Altenburg 2C1	48.1333	16.9		Böhme and Ilg 2003
Vipera ammodytes	V. ammodytes	1	1.8	Italy	Pietrafitta (Umbria)	43	12.2		Böhme and Ilg 2003
Vipera ammodytes	V. ammodytes	2.6	3.6	Poland	Rebielice Krolewskie 1A	51	18.8667		Böhme and Ilg 2003
Vipera ammodytes	V. ammodytes	2.8	3.6	Poland	Rebielice Krolewskiee 2	51	18.8667		Böhme and Ilg 2003
Vipera ammodytes	V. ammodytes	3.6	4.2	Poland	Weze 1	52.35	22.15		Böhme and Ilg 2003
Vipera ammodytes	V. ammodytes	5	5.33	Hungary	Osztramos 1C	48.525	20.7583		Böhme and Ilg 2003
Vipera aspis	Vipera aspis	0	0.01	Italy	Grotta dell'Edera, Aurisina	45.7475	13.6786		Böhme and Ilg 2003
Vipera aspis	Vipera aspis	0	0.05	Italy	San Vito Lo Capo K22, Sicily	38.2	12.75		Böhme and Ilg 2003

Fossil	Phylogenetic affinity	Min age	Max age	Country	Locality	Lat	Long	Notes	Reference
Vipera aspis	Vipera aspis	0.03	0.038	Spain	Teixoneres II Cave	41.8	1.9		Böhme and Ilg 2003
Vipera aspis	Vipera aspis	0.031	0.038	Italy	Grotta Generosa, San Fedele Intelvi, Como	45.93	9.02		Böhme and Ilg 2003
Vipera aspis	Vipera aspis	0.04	0.07	Spain	Abric Romani near Capellades, 45 km NW Barcelona	41.5328	1.6881		Böhme and Ilg 2003
Vipera aspis	Vipera aspis	0	0.127	Italy	Gabrovizza	45.7264	13.7294		Böhme and Ilg 2003
Vipera aspis	Vipera aspis	0.01	0.127	France	Cantet Cave, Espeche, Hautes Pyrenees	43.05	0.3		Böhme and Ilg 2003
Vipera aspis	Vipera aspis	0.08	0.127	France	Artenac, Charente	45.85	0.3333		Böhme and Ilg 2003
Vipera aspis	Vipera aspis	0.1	0.11	Italy	Valdemino Cave, 26-27 (Borgio Verezzi, Liguria)	44.1633	12.4523		Böhme and Ilg 2003
Vipera aspis	Vipera aspis	0.12	0.2	France	Lazaret C, Alpes Maritimes				Böhme and Ilg 2003
Vipera aspis	Vipera aspis	0.4	0.4	Spain	Gran Dolina TD 10, Atapuerca, Burgos	42.34	-3.44		Böhme and Ilg 2003
Vipera aspis	Vipera aspis	0.127	0.7	France	Montousse 4, Hautes Pyrenees	43.0667	0.4167		Böhme and Ilg 2003

Fossil	Phylogenetic affinity	Min age	Max age	Country	Locality	Lat	Long	Notes	Reference
Vipera aspis	Vipera aspis	0.2	0.7	Italy	Riparo di Visogliano	45.78	13.65		Böhme and Ilg 2003
Vipera aspis	Vipera aspis	0.126	0.781	Spain	Cova d'Olopte (Cova B), Isovol, Cerdanya	42.3866	1.493		Böhme and Ilg 2003
Vipera aspis	Vipera aspis	0.4	0.9	Spain	Gran Dolina, Atapuerca, Burgos	42.34	-3.44		Böhme and Ilg 2003
Vipera aspis	Vipera aspis	0.7	0.7	Italy	Valdemino Cave, 20-24 (Borgio Verezzi, Liguria)	44.1633	12.4523		Böhme and Ilg 2003
Vipera aspis	Vipera aspis	0.85	0.85	Spain	Gran Dolina TD 6-1+TD 6-2, Atapuerca, Burgos	42.34	-3.44		Böhme and Ilg 2003
Vipera aspis	Vipera aspis	0.85	0.9	Spain	Gran Dolina TD 6-3, Atapuerca, Burgos	42.34	-3.44		Böhme and Ilg 2003
Vipera aspis	Vipera aspis	0.012	1.806	Spain	L´Escala, Cala Montgo, Girona	41.95	3.2		Böhme and Ilg 2003
Vipera aspis	Vipera aspis	0.7	1.64	Italy	Cava Dell'Erba Apricena, Foggia	41.45	15.5667		Böhme and Ilg 2003
Vipera aspis	Vipera aspis	0.781	1.806	Spain	Bagur-2 (B-2), Begur, Girone	41.95	3.2167		Böhme and Ilg 2003
Vipera aspis	Vipera aspis	1.3	1.7	Italy	Pirro Nord (Cava dell´Erba, Cava Pirro), Apricena, Apulia	41.8019	15.3847		Böhme and Ilg 2003

Fossil	Phylogenetic affinity	Min age	Max age	Country	Locality	Lat	Long	Notes	Reference
Vipera aspis	Vipera aspis	1.8	1.9	Spain	Casablanca 1 (ACB-1), Almenara, Castellon	39.75	-0.2167		Böhme and Ilg 2003
Vipera aspis	Vipera aspis	1.9	2	France	Montousse 5, Hautes Pyrenees	43.0667	0.4167		Böhme and Ilg 2003
Vipera aspis	Vipera aspis	2	3	Spain	Zujar, Andalucia	37.54	-2.8434		Böhme and Ilg 2003
Vipera aspis	Vipera aspis	2.9	3	France	Mas Genegals, Herault	42.85	2.8		Böhme and Ilg 2003
Vipera natiensis	sister to Vipera aspis	3.6	5.3	Spain	Punta Nati near Ciutadella, Minorca	40.0506	3.8257	The vertebrae of Vipera natiensis appear to be close to those of Vipera aspis	Bailon et al. 2002
Vipera natiensis	sister to Vipera aspis	5	5.33	Spain	Calo d´en Rafelino, Manacor, Mallorca	39.5566	3.3647	The vertebrae of Vipera natiensis appear to be close to those of Vipera aspis	Bailon et al. 2002
Vipera aspis group	A non-monophyletic group of: V. aspis,V. latastei, V. monticola, V. ammodytes, V. transcaucasiana	9.9	10.3	Hungary	Rudabanya	48.3833	20.6333		Szyndlar and Rage 2002
Vipera aspis group	A non-monophyletic group of: V. aspis,V. latastei, V. monticola, V. ammodytes, V. transcaucasiana	11.2	11.4	Hungary	Felsotarkany	47.9667	20.4167		Venczel 2011
Vipera aspis group	A non-monophyletic group of: V. aspis,V. latastei, V. monticola, V. ammodytes, V. transcaucasiana	11.6	11.8	Hungary	Felsotarkany	47.9566	20.3937		Venczel 2011
Vipera aspis group	A non-monophyletic group of: V. aspis,V. latastei, V. monticola, V. ammodytes, V. transcaucasiana	12.1	13.1	France	La-Grive-Saint-Alban (M+L7), Ilsere	45.58	5.26		Szyndlar and Rage 2002

Fossil	Phylogenetic affinity	Min age	Max age	Country	Locality	Lat	Long	Notes	Reference
Vipera aspis group	A non-monophyletic group of: V. aspis,V. latastei, V. monticola, V. ammodytes, V. transcaucasiana	13	14	Romania	Subpiatraƒ 2/1R, Bihor District	47	22.3		Venczel 2011
Vipera aspis group	A non-monophyletic group of: V. aspis,V. latastei, V. monticola, V. ammodytes, V. transcaucasiana	13.5	13.7	Slovakia	Devinska Nova Ves - Bonanza	48.0167	16.9333		Ivanov 1998
Vipera aspis group	A non-monophyletic group of: V. aspis,V. latastei, V. monticola, V. ammodytes, V. transcaucasiana	14	17.5	France	Vieux Collonges	45.75	4.85	The neural spine is not so extremely low as it is in case of representatives of the complex 'berus', therefore, it is assumed that these vertebrae belong to the representatives of the complex 'aspis'	Ivanov 2000
Vipera aspis group	A non-monophyletic group of: V. aspis,V. latastei, V. monticola, V. ammodytes, V. transcaucasiana	14.8	17	Germany	Rothenstein 1/13	48.967	11.059		Szyndlar 2009
Vipera aspis group	A non-monophyletic group of: V. aspis,V. latastei, V. monticola, V. ammodytes, V. transcaucasiana	17	17.5	France	Bezian, La Romieu, Gers	44.2	9		Szyndlar and Rage 2002
Vipera aspis group	A non-monophyletic group of: V. aspis,V. latastei, V. monticola, V. ammodytes, V. transcaucasiana	17.6	17.6	Austria	Oberdorf N Voitsberg, Opencast Mine (O3+O4)	47.0667	15.1167		Szyndlar and Rage 2002
Vipera aspis group	A non-monophyletic group of: V. aspis,V. latastei, V. monticola, V. ammodytes, V. transcaucasiana	17.3	18	France	Beon 1	43.95	0.2	The vertebrae are not elongate, which rules out a referral to the 'berus complex'. The overall proportions are consistent with those in the 'Vipera aspis complex'.	Rage and Bailon 2006
Vipera aspis group	A non-monophyletic group of: V. aspis,V. latastei, V. monticola, V. ammodytes, V. transcaucasiana	17.5	18	France	Artenay (Loiret)	48.1	1.9		Szyndlar and Rage 2002
Vipera aspis group	A non-monophyletic group of: V. aspis,V. latastei, V. monticola, V. ammodytes, V. transcaucasiana	17.5	20	Spain	Agramon	38.4167	-1.6333		Szyndlar and Rage 2002
Vipera aspis group	A non-monophyletic group of: V. aspis,V. latastei, V. monticola, V. ammodytes, V. transcaucasiana	20	20	Czech Republic	Merkur North, opencast mine, Ahnikov	50.45	13.4333	The vertebrae are small with low ratio cl/naw; therefore, they were assigned to the 'Vipera aspis complex'	Ivanov 2002

Fossil	Phylogenetic affinity	Min age	Max age	Country	Locality	Lat	Long	Notes	Reference
Vipera aspis group	A non-monophyletic group of: V. aspis,V. latastei, V. monticola, V. ammodytes, V. transcaucasiana	20.5	21	France	Laugnac (Lot-et-Garonne)	44.247	0.4502		Szyndlar and Rage 2002
Vipera aspis group	A non-monophyletic group of: V. aspis,V. latastei, V. monticola, V. ammodytes, V. transcaucasiana	21	22	France	Saint-Gerand-le-Puy, Allier	46.2581	3.512		Szyndlar and Rage 2002
Vipera aspis group	A non-monophyletic group of: V. aspis,V. latastei, V. monticola, V. ammodytes, V. transcaucasiana	4.85	5.23	Poland	Podlesice	50.5667	19.5333		Szyndlar and Rage 2002
Vipera aspis group	A non-monophyletic group of: V. aspis,V. latastei, V. monticola, V. ammodytes, V. transcaucasiana	5	6	Italy	Gargano	41.8	15.4	The general shape of the vertebral body and hypapophysis indicate the presence of the V. aspis group	Delfino and Bailon 2000
Vipera aspis group	A non-monophyletic group of: V. aspis,V. latastei, V. monticola, V. ammodytes, V. transcaucasiana	5.35	6.15	Hungary	Polgardi	47.05	18.3		Szyndlar 1991
Vipera aspis group	A non-monophyletic group of: V. aspis,V. latastei, V. monticola, V. ammodytes, V. transcaucasiana	5.33	6.2	Hungary	Polgardi	47.05	18.3		Szyndlar 1991
Vipera aspis group	A non-monophyletic group of: V. aspis,V. latastei, V. monticola, V. ammodytes, V. transcaucasiana	8.7	9.2	Ukraine	Krivoj Rog	47.9167	33.35		Szyndlar and Rage 2002
Vipera meotica	A non-monophyletic group of: V. aspis,V. latastei, V. monticola, V. ammodytes, V. transcaucasiana	5.332	7.246	Ukraine	Orekhovka	48.2867	39.2038	A member of the aspis complex	Szyndlar and Rage 2002
Vipera meotica	A non-monophyletic group of: V. aspis,V. latastei, V. monticola, V. ammodytes, V. transcaucasiana	7	7.6	Ukraine	Cherevichnoje lower level	46.6333	30.6167	A member of the aspis complex	Szyndlar and Rage 2002
Vipera berus	Vipera berus	0.003	0.007	England	Creag nan Uamh Caves, Badger Cave, Assynt, north-west Scotland	58.1084	-4.9391		Böhme and Ilg 2003

Fossil	Phylogenetic affinity	Min age	Max age	Country	Locality	Lat	Long	Notes	Reference
Vipera berus	Vipera berus	0.001	0.01	Germany	Gamsenberg (Vorderer) near Oppurg, Thuringia	50.7167	11.65		Böhme and Ilg 2003
Vipera berus	Vipera berus	0.004	0.008	England	Creag nan Uamh Caves, Bone Cave, Assynt, north-west Scotland	58.1084	-4.9391		Böhme and Ilg 2003
Vipera berus	Vipera berus	0.005	0.012	England	Torbryon Caves, Broken Cavern, Devon	50.4944	-3.6706		Böhme and Ilg 2003
Vipera berus	Vipera berus	0.01	0.01	England	Cow Cave, Chudleigh, Devonshire	50.6063	-3.62		Böhme and Ilg 2003
Vipera berus	Vipera berus	0.01	0.1	Germany	Saalfeld Roter Berg, Thuringia	50.65	11.3667		Böhme and Ilg 2003
Vipera berus	Vipera berus	0.005	0.127	Germany	Dietfurt near Sigmaringen	48.0667	9.1333		Böhme and Ilg 2003
Vipera berus	Vipera berus	0.01	0.127	Germany	Burgtonna, Deckschichten	51.0667	10.7333		Böhme and Ilg 2003
Vipera berus	Vipera berus	0.075	0.085	France	Bois Roche Cave 1a, near Cognac, Charente	45.7	-0.3333		Blain and Villa 2006
Vipera berus	Vipera berus	0.12	0.2	France	Lazaret C, Alpes Maritimes				Böhme and Ilg 2003
Vipera berus	Vipera berus	0.007	0.4	England	Kent´s Cavern (Wolf Den), Wellswood, 2km E Torquay, Devon	50.4682	-3.503		Böhme and Ilg 2003

**Fossil**	**Phylogenetic affinity**	**Min age**	**Max age**	**Country**	**Locality**	**Lat**	**Long**	**Notes**	**Reference**
Vipera berus	Vipera berus	0.374	0.424	England	East Farm, Barnham, Suffolk	52.3667	52.3667		Böhme and Ilg 2003
Vipera berus	Vipera berus	0.127	0.7	Austria	St. Margarethen, near Hainburg	47.8	16.6		Böhme and Ilg 2003
Vipera berus	Vipera berus	0.7	0.7	Poland	Kozi Grzbiet	50.7964	15.3844		Böhme and Ilg 2003
Vipera berus	Vipera berus	0.7	1.64	Czech Republic	Mala Dohoda Quarry, Moravian Karst Area				Böhme and Ilg 2003
Vipera berus	Vipera berus	0.78	1.8	Poland	Jaskinia Zabia				Böhme and Ilg 2003
Vipera berus	Vipera berus	2	2.2	Poland	Kadzielnia	50.2036	20.9278		Böhme and Ilg 2003
Vipera berus group	A clade of: V. berus, ursinii, and all other related species	1.9	2	France	Montousse 5, Hautes Pyrenees	43.0667	0.4167	Previous understanding of the V. berus group included also V. ursinii and related species	Grooombridge 1986<br>Szyndlar 1991<br>Szyndlar and Rage 2002
Vipera berus group	A clade of: V. berus, ursinii, and all other related species	2.6	3.6	Greece	Tourkobounia 1	37.99	23.49	Previous understanding of the V. berus group included also V. ursinii and related species	Grooombridge 1986<br>Szyndlar 1991<br>Szyndlar and Rage 2002
Vipera berus group	A clade of: V. berus, ursinii, and all other related species	3.1	3.3	Spain	Las Higueruelas, Alcolea de Calatrava, Ciudad Real	38.9883	-4.0857	Previous understanding of the V. berus group included also V. ursinii and related species	Grooombridge 1986<br>Szyndlar 1991<br>Szyndlar and Rage 2002
Vipera berus group	A clade of: V. berus, ursinii, and all other related species	5	5.33	Hungary	Osztramos 1C	48.525	20.7583	Previous understanding of the V. berus group included also V. ursinii and related species	Grooombridge 1986<br>Szyndlar 1991<br>Szyndlar and Rage 2002

Fossil	Phylogenetic affinity	Min age	Max age	Country	Locality	Lat	Long	Notes	Reference
Vipera berus group	A clade of: V. berus, ursinii, and all other related species	5.5	7	Ukraine	Andreevka	48.0167	37.05	Previous understanding of the V. berus group included also V. ursinii and related species	Grooombridge 1986<br>Szyndlar 1991<br>Szyndlar and Rage 2002
Vipera berus group	A clade of: V. berus, ursinii, and all other related species	7.28	7.38	Ukraine	Novaya Emetovka 2 (Kostev ravine)	46.7333	30.5333	Previous understanding of the V. berus group included also V. ursinii and related species	Grooombridge 1986<br>Szyndlar 1991<br>Szyndlar and Rage 2002
Vipera berus group	A clade of: V. berus, ursinii, and all other related species	7.6	8.7	Ukraine	Frunzovka 2	47.333	29.75	Previous understanding of the V. berus group included also V. ursinii and related species	Grooombridge 1986<br>Szyndlar 1991<br>Szyndlar and Rage 2002
Vipera berus group	A clade of: V. berus, ursinii, and all other related species	8.1	8.8	Ukraine	Novoelizavetovka 2+3, lower layer	47.15	30.4055	Previous understanding of the V. berus group included also V. ursinii and related species	Grooombridge 1986<br>Szyndlar 1991<br>Szyndlar and Rage 2002
Vipera latastei	Vipera latastei	0.013	0.015	England	Gorham´s Cave IIIa, Gibraltar Peninsula	36.1203	-5.3419		Böhme and Ilg 2003
Vipera latastei	Vipera latastei	0.017	0.023	England	Gorham´s Cavee IIIb, Gibraltar Peninsula	36.1203	-5.3419		Böhme and Ilg 2003
Vipera latastei	Vipera latastei	0.027	0.039	England	Gorham´s Cave IV, Gibraltar Peninsula	36.1203	-5.3419		Böhme and Ilg 2003
Vipera latastei	Vipera latastei	0.021	0.057	Spain	Higueral de Valleja Cave	36.75	-5.81		Böhme and Ilg 2003
Vipera latastei	Vipera latastei	0.047	0.048	Spain	Cova dels Xaragalls layer C4, Vimbodi-Poblet, Tarragona, Catalonia	41.008	1.0488		Böhme and Ilg 2003
Vipera latastei	Vipera latastei	0.054	0.055	Spain	Cova dels Xaragalls layer C6, Vimbodi-Poblet, Tarragona, Catalonia	41.008	1.0488		Böhme and Ilg 2003

Fossil	Phylogenetic affinity	Min age	Max age	Country	Locality	Lat	Long	Notes	Reference
Vipera latastei	Vipera latastei	0.01	0.127	Spain	Cueva Hora (Darro, Granada)	37.35	-3.3		Böhme and Ilg 2003
Vipera latastei	Vipera latastei	0.068	0.081	Spain	Cueva del Camino Secteur Sud, Pinilla del Valle, Madrid	40.9254	-3.8063		Böhme and Ilg 2003
Vipera latastei	Vipera latastei	0.079	0.089	Spain	Hat, Jarama valley, San Martin de la Vega, 18 km SE Madrid	40.2616	-3.5347		Böhme and Ilg 2003
Vipera latastei	Vipera latastei	0.083	0.099	Spain	Cueva del Camino Secteur Central, Pinilla del Valle, Madrid	40.9254	-3.8063		Böhme and Ilg 2003
Vipera latastei	Vipera latastei	0.084	0.1	Spain	Cueva del Camino Secteur Nord, Pinilla del Valle, Madrid	40.9254	-3.8063		Böhme and Ilg 2003
Vipera latastei	Vipera latastei	0.25	0.35	Spain	Sima del Elefante TE18+TE19, Sierra de Atapuerca, Burgos	42.33	-3.51		Böhme and Ilg 2003
Vipera latastei	Vipera latastei	0.011	0.7	Spain	Cueva del Agua, Iznalloz, Granada	36.9333	-1.9333		Böhme and Ilg 2003
Vipera latastei	Vipera latastei	0.4	0.4	Spain	Gran Dolina TD 10, Atapuerca, Burgos	42.34	-3.44		Blain et al. 2008
Vipera latastei	Vipera latastei	0.4	0.9	Spain	Gran Dolina (TD=Trinchera Dolina), Atapuerca, Burgos	42.34	-3.44		Blain et al. 2008
Vipera latastei	Vipera latastei	1.1	1.1	Spain	Casablanca 3 (ACB-3), Almenara, Castellon	39.75	-0.2167		Blain et al. 2007

**Fossil**	**Phylogenetic affinity**	**Min age**	**Max age**	**Country**	**Locality**	**Lat**	**Long**	**Notes**	**Reference**
Vipera latastei	Vipera latastei	1.1	1.2	Spain	Cueva de la Victoria-1 (CV-1), Carthagene, Murcia	37.6167	-0.8667		Böhme and Ilg 2003
Vipera latastei	Vipera latastei	0.8	1.8	Spain	El Chaparral, Villaluenga del Rosario, Cadiz	36.6982	-5.3888		Böhme and Ilg 2003
Vipera latastei	Vipera latastei	1.8	1.9	Spain	Casablanca 1 (ACB-1), Almenara, Castellon	39.75	-0.2167		Böhme and Ilg 2003
Vipera latastei	Vipera latastei	1.85	1.9	Spain	Vallirana (VA), Castelldelfs, Massif de Garraf, Barcelone	41.8333	1.9333		Böhme and Ilg 2003
Vipera latastei	Vipera latastei	2.3	2.35	Spain	Casablanca 4 (ACB-4), Almenara, Castellon	39.75	-0.2167		Böhme and Ilg 2003
Vipera ursinii	Vipera ursinii	0.12	0.2	France	Lazaret C, Alpes Maritimes				Böhme and Ilg 2003
Vipera ursinii	Vipera ursinii	0.13	0.38	Russia	Chernyi Yar-Nizhnee Zaimishche, Volgograd	48.0606	46.1162		Böhme and Ilg 2003
Vipera ursinii	Vipera ursinii	0.7	1.64	Czech Republic	Stranska Skala Hill near Brno	49.2	16.6333		Böhme and Ilg 2003
Vipera ursinii	Vipera ursinii	2	2.2	Ukraine	Kotlovina, middle level	45.5	28.5667		Szyndlar and Rage 2002
Vipera ursinii	Vipera ursinii	2.2	2.5	Ukraine	Tikhonovka 1 & 2	46.0167	33.7833		Böhme and Ilg 2003

Fossil	Phylogenetic affinity	Min age	Max age	Country	Locality	Lat	Long	Notes	Reference
Vipera antiqua	Vipera	17	17.5	Czech Republic	Dolnice near Cheb	50.0833	12.3833	The oldest known member of the genus Vipera	Szyndlar 1987<br>Szyndlar and Rage 2002
Vipera antiqua	Vipera	17.5	18	Germany	Petersbuch 2	48.9779	11.1909	The oldest known member of the genus Vipera	Szyndlar 1987<br>Szyndlar and Rage 2002
Vipera antiqua	Vipera	20.5	22	Germany	Hessler, Wiesbaden-Biebrich	50.25	7.75	The oldest known member of the genus Vipera	Szyndlar 1987<br>Szyndlar and Rage 2002
Vipera antiqua	Vipera	21.5	22.5	Germany	Mainz-Weisenau (quarry, Fp.19)	49.9075	8.298	The oldest known member of the genus Vipera	Szyndlar 1987<br>Szyndlar and Rage 2002
Vipera maghrebiana	Vipera	13	13.1	Morocco	Beni Mellal	32.5	-6.5	This snake closely resembles smaller members of the genus Vipera (European vipers)	Szyndlar and Rage 2002
Vipera sp.	Vipera	0.004	0.015	Spain	Valdavara-1 cave, Lugo	42.85	-7.16		Böhme and Ilg 2003
Vipera sp.	Vipera	0.017	0.037	Spain	El PortalĂłn, entrance Cueva Mayor, Sierra Atapuerca, Burgos	42.33	-3.51		Böhme and Ilg 2003
Vipera sp.	Vipera	0.031	0.039	Spain	Cueva del Conde, Santo Adriano, Asturias	43.3167	-5.9833		Böhme and Ilg 2003
Vipera sp.	Vipera	0.039	0.04	Spain	Canyars in the town of GavĂ , 20 km W Barcelona	41.4161	2.231		Böhme and Ilg 2003
Vipera sp.	Vipera	0.005	0.127	Germany	Malerfels near Gredinge, Nuremberg area	49.0483	11.3614		Böhme and Ilg 2003

Fossil	Phylogenetic affinity	Min age	Max age	Country	Locality	Lat	Long	Notes	Reference
Vipera sp.	Vipera	0.011	0.126	Spain	Cova del Gegant, Massif of Garraf, Sitges, Barcelona	41.2333	1.7333		Böhme and Ilg 2003
Vipera sp.	Vipera	0.074	0.147	Spain	Cova del Rinoceront, eastern Garraf Massif, CanÂ´Aymerich quarry, Castelldelfs	41.2736	1.9609		Böhme and Ilg 2003
Vipera sp.	Vipera	0	0.45	Ukraine	Ozyornoye-1 near Ozernoe, east bank of Jalpug Lake, Odessa Region	45.4	28.6667		Böhme and Ilg 2003
Vipera sp.	Vipera	0.39	0.41	Turkey	Emirkaya 2, 1.5 km south of Seydisehir	37.405	31.8346		Böhme and Ilg 2003
Vipera sp.	Vipera	0.127	0.7	Croatia	Dubci				Böhme and Ilg 2003
Vipera sp.	Vipera	0.475	0.51	Russia	Kuznetsovka left board of Podgornyi Buerak, Uvarovo rayon, Tambov oblast`	51.96	42.19		Böhme and Ilg 2003
Vipera sp.	Vipera	0.5	0.65	Russia	Koziy Ovrag	50.9	39.8		Böhme and Ilg 2003
Vipera sp.	Vipera	0.7	0.8	Russia	Ilyinka 2	52	42		Böhme and Ilg 2003
Vipera sp.	Vipera	0.01	1.64	Germany	Stuttgart Viadukt	48.7667	9.1833		Böhme and Ilg 2003
Vipera sp.	Vipera	1	1.1	Moldova	Chishmikioi	45.533	28.3888		Böhme and Ilg 2003

Fossil	Phylogenetic affinity	Min age	Max age	Country	Locality	Lat	Long	Notes	Reference
Vipera sp.	Vipera	0.7	1.64	France	Mas Rambault near Montpellier	43.5	3.6		Böhme and Ilg 2003
Vipera sp.	Vipera	1.06	1.38	Spain	Sima del Elefante TE9, Sierra de Atapuerca, Burgos	42.33	-3.51		Böhme and Ilg 2003
Vipera sp.	Vipera	0.8	1.8	Italy	Monte Argentario (Miniera della Polveriera), Grosseto	42.4065	11.1545		Böhme and Ilg 2003
Vipera sp.	Vipera	1.3	1.4	Spain	Sierra de Quibas, Abanilla, Murcia	38.3	-1.05		Böhme and Ilg 2003
Vipera sp.	Vipera	1.5	2.5	Italy	Monte Tuttavista Cava VI (3) - antica, Sardinia	40.3833	9.7		Böhme and Ilg 2003
Vipera sp.	Vipera	2.2	2.5	Ukraine	Bolshevik 2	50.45	30.44		Böhme and Ilg 2003
Vipera sp.	Vipera	2.5	2.6	Spain	Cova Bonica (CBo), Massif of Garraf, GavĂ , Barcelona	41.38	1.93		Böhme and Ilg 2003
Vipera sp.	Vipera	2.6	2.7	Ukraine	Zhevakhova Gora	46.55	30.7333		Böhme and Ilg 2003
Vipera sp.	Vipera	2.5	3.5	Moldova	Novye Tanatary	46.7165	29.4165		Böhme and Ilg 2003
Vipera sp.	Vipera	2.588	3.6	Italy	Capo Mannu D1 Local Fauna (Mandriola), Sardinia	40.0409	8.3845		Böhme and Ilg 2003

Fossil	Phylogenetic affinity	Min age	Max age	Country	Locality	Lat	Long	Notes	Reference
Vipera sp.	Vipera	2.6	3.6	Ukraine	Vinogradovka	45.8333	29.3833		Böhme and Ilg 2003
Vipera sp.	Vipera	2.8	3.6	Austria	Bad Deutsch-Altenburg 20	48.1333	16.9		Böhme and Ilg 2003
Vipera sp.	Vipera	3.5	3.6	Spain	Layna, Province de Soria	41.1	-2.3		Böhme and Ilg 2003
Vipera sp.	Vipera	3.6	4.2	Moldova	Musaid right bank of Big Salcha River, Vulkaneshty Region	45.8206	28.505		Böhme and Ilg 2003
Vipera sp.	Vipera	3.6	5.3	Spain	Vilafant, Girona	42.2492	2.9378		Böhme and Ilg 2003
Vipera sp.	Vipera	4	7	Spain	Na Burguesa-1, Mallorca	39.5704	2.5579		Böhme and Ilg 2003
Vipera sp.	Vipera	5.5	7	Italy	Cava Passalacqua near Apricena	41.8	15.4		Böhme and Ilg 2003
Vipera sp.	Vipera	6.8	7.7	Hungary	Tardosbanya 3	47.6667	18.45		Venczel 1998
Vipera sp.	Vipera	9.7	11.1	Spain	Hostalets de Pierola, Catalonia, Spain	41.5349	1.7685		Carmona et al. 2010
Vipera sp.	Vipera	11.04	12.014	Ukraine	Gritsev (Khmelnitsk area, Shepetovski district)	49.975	27.16		Szyndlar 1991

Fossil	Phylogenetic affinity	Min age	Max age	Country	Locality	Lat	Long	Notes	Reference
Vipera sp.	Vipera	13.5	13.7	Slovakia	Devinska Nova Ves - Bonanza	48.0167	16.9333	Belongs to the 'European vipers' group of the uncertain complex	Szyndlar 1991
Vipera sp.	Vipera	16.4	17.25	Germany	Randecker Maar	48.5667	9.5333		Szyndlar and Rage 2002
Vipera sp.	Vipera	17.75	17.8	Germany	Langenau 1	48.5003	10.1219		Szyndlar and Rage 2002
Vipera sp.	Vipera	20.43	23.03	Italy	Oschiri, Sardinia	40.7222	9.0973	The short vertebral centrum somewhat recalls the morphology of V. aspis, however, due to the scarcity of the material and its preservation, the identification is limited at genus rank	Delfino et al. 2011
Vipera sensu lato	A clade of: Vipera, Daboia, Montivipera, Macrovipera	12.75	13.25	Germany	Kleineisenbach	48.3639	11.5988	Could not be assigned to a genus	Szyndlar and Rage 2002
Vipera sensu lato	A clade of: Vipera, Daboia, Montivipera, Macrovipera	14	16	France	Pontigne-les-Buisseneaux, Maine-et-Loire	47.54	-0.0401	Could not be assigned to a genus	Szyndlar and Rage 2002
Vipera sensu lato	A clade of: Vipera, Daboia, Montivipera, Macrovipera	17.75	17.8	Germany	Langenau 1	48.5003	10.1219	Could not be assigned to a genus	Szyndlar and Rage 2002
Vipera sensu lato	A clade of: Vipera, Daboia, Montivipera, Macrovipera	18	20	Germany	Stubersheim 3	48.5947	9.9139	Could not be assigned to a genus	Szyndlar and Rage 2002

Supplementary Table S4. Stem and crown age estimates (Ma) of the viperine genera based on the FBD calibration. *Eristicophis* and *Proatheris* are monotypic and hence do not have crown age estimates.

	Stem age		Crown age	
**Genus**	**Mean**	**95% HPD**	**Mean**	**95% HPD**
Atheris	27.7	24.2–31.4	13.4	10.8–16.2
Bitis	27.7	24.2–31.4	23.5	20.3–26.8
Causus	34.0	29.2–37.8	18.6	15.2–22.1
Cerastes	28.9	25.1–32.8	15.3	11.6–19.1
Daboia	20.2	17.9–22.6	16.1	13.6–18.8
Echis	28.9	25.1–32.8	18.9	16.4–21.7
Eristicophis	18.0	14.4–21.6	na	na
Macrovipera	12.6	11.9–13.8	7.2	5.1–9.2
Montivipera	12.6	11.9–13.8	7.3	6.3–8.4
Proatheris	28.6	24.8–32.6	na	na
Pseudocerastes	18.0	14.4–21.6	12.0	8.6–15.2
Vipera	20.2	17.9–22.6	13.4	11.5–15.4

Supplementary Table S5. Results of the biogeographic reconstructions using BioGeoBEARS. Statistics for the competing models and estimated parameter values are shown. The best models as identified by AICc are in bold.

Input tree	Model	LnL	No. of parameters	Dispersal rate (d)	Extinction rate (e)	Founder-event speciation (j)	AICc	AIC_wt
Viperinae (83 tips)	DEC	-86.53	2	0.0045	1.00E-12	-	177.07	2.04E-06
	**DEC+J**	**-72.69**	**3**	**0.0013**	**1.00E-12**	**0.0310**	**151.38**	**7.74E-01**
	DIVALIKE	-87.25	2	0.0067	1.00E-12	-	178.50	9.95E-07
	DIVALIKE+J	-74.02	3	0.0018	1.00E-12	0.0339	154.04	2.04E-01
	BAYAREALIKE	-115.79	2	0.0025	3.41E-02	-	235.58	4.01E-19
	BAYAREALIKE+J	-76.23	3	0.0011	1.00E-07	0.0380	158.47	2.23E-02
Viperinae + Azemiopinae + Crotalinae (282 tips)	DEC	-151.86	2	0.3044	1.00E-12	-	307.76	8.59E-03
	**DEC+J**	**-146.09**	**3**	**0.1936**	**1.00E-12**	**0.0032**	**298.26**	**9.91E-01**
	DIVALIKE	-164.57	2	0.4332	1.00E-12	-	333.19	2.58E-08
	DIVALIKE+J	-155.85	3	0.2664	1.00E-12	0.0048	317.79	5.72E-05
	BAYAREALIKE	-262.11	2	0.1612	2.55E-01	-	528.27	1.12E-50
	BAYAREALIKE+J	-164.90	3	0.1195	1.32E-01	0.0076	335.88	6.72E-09

References for Supplementary Tables

Bachmayer, F. & Szyndlar, Z. (1987) A second contribution to the ophidian fauna (Reptilia: Serpentes) of Kohfidisch, Austria. *Annalen des Naturhistorischen Museums in Wien, 88*, 25–39.

Bailon, S. (1991) Amphibiens et reptiles du pliocène et du quaternaire de France et d'Espagne: mise en place et évolutioin des faunes. *In*. Université Paris, Paris.

Bailon, S., Bover, P., Quintana, J. & Alcover, J.A. (2010) First fossil record of *Vipera* Laurenti 1768 "Oriental vipers complex"(Serpentes: Viperidae) from the Early Pliocene of the western Mediterranean islands. *Comptes Rendus Palevol, 9*, 147–154.

Bailon, S., Garcia-Porta, J. & Quintana-Cardona, J. (2002) Première découverte de Viperidae (Reptilia, Serpentes) dans les îles Baléares (Espagne): des vipères du Néogène de Minorque. Description d'une nouvelle espèce du Pliocène. *Comptes Rendus Palevol, 1*, 227–234.

Barlow, A., W. Grail, M. de Bruyn, and W. Wüster. 2012. Anonymous nuclear markers for the African adders (Serpentes: Viperidae: *Bitis*). Conservation Genetics Resources 4:967–969.

Blain, H.-A., Bailon, S. & Agusti, J. (2007) Anurans and squamate reptiles from the latest early Pleistocene of Almenara-Casablanca-3 (Castellón, East of Spain). Systematic, climatic and environmental considerations. *Geodiversitas, 29*, 269–295.

Blain, H.-A., Bailon, S. & Cuenca-Bescós, G. (2008) The Early-Middle Pleistocene palaeoenvironmental change based on the squamate reptile and amphibian proxies at the Gran Dolina site, Atapuerca, Spain. *Palaeogeography, Palaeoclimatology, Palaeoecology, 261*, 177–192.

Blain, H.-A. & Villa, P. (2006) Amphibians and squamate reptiles from the early Upper Pleistocene of Bois Roche Cave (Charente, southwestern France). *Acta Zoologica Cracoviensia-Series A: Vertebrata, 49*, 1–32.

Böhme, M. & Ilg, A. (2003) FosFARbase. *Accessed June 2017*, 2007.

Burbrink, F. T., R. Lawson, and J. B. Slowinski. 2000. Mitochondrial DNA phylogeography of the polytypic North American rat snake (*Elaphe obsoleta*): a critique of the subspecies concept. Evolution 54:2107–2118.

Delfino, M. & Bailon, S. (2000) Early Pleistocene herpetofauna from Cava dell'Erba and Cava Pirro (Apulia, southern Italy). *Herpetological Journal, 10*, 95–110.

Delfino, M., Bailon, S. & Pitruzzella, G. (2011) The Late Pliocene amphibians and reptiles from "Capo Mannu D1 Local Fauna"(Mandriola, Sardinia, Italy). *Geodiversitas, 33*, 357–382.

Geraads, D. (2006) The late Pliocene locality of Ah1 a1 Oughlam, Morocco: vertebrate fauna and interpretation. *Transactions of the Royal Society of South Africa, 61*, 97–101.

Groombridge, B. (1986) Phyletic relationships among viperine snakes. *In:* Roček, Z. (Ed), *Studies in Herpetology*, Prague, pp. 219–222.

Ivanov, M. (1998) The snake fauna of Devínska Nová Ves (Slovak Republic) in relation to the evolution of snake assemblages of the European Middle Miocene. *Acta Musei Moraviae, Scientiae Geologicae, 53*, 159–172.

Ivanov, M. (2000) Snakes of the lower/middle Miocene transition at Vieux Collonges (Rhône, France), with comments on the colonisation of western Europe by colubroids. *Geodiversitas, 22*, 559–588.

Ivanov, M. (2002) The oldest known Miocene snake fauna from Central Europe: Merkur-North locality, Czech Republic. *Acta Palaeontologica Polonica, 47*, 513–534.

Ivanov, M. & Böhme, M. (2011) Snakes from Griesbeckerzell (Langhian, Early Badenian), North Alpine Foreland Basin (Germany), with comments on the evolution of snake faunas in Central Europe during the Miocene Climatic Optimum. *Geodiversitas, 33*, 411–449.

Lawson, R., J. B. Slowinski, B. I. Crother, and F. T. Burbrink. 2005. Phylogeny of the Colubroidea (Serpentes): new evidence from mitochondrial and nuclear genes. Mol. Phylogen. Evol. 37:581-601.

Rage, J.-C. (1973) Fossil snakes from Olduvai, Tanzania. *Fossil vertebrates of Africa, 3*, 1–6.

Rage, J.-C. (2003) Squamate reptiles from the early Miocene of Arrisdrift (Namibia). *Geology and Palaeobiology of the central and southern Namib, 2*, 43–50.

Rage, J.-C. & Bailon, S. (2005) Amphibians and squamate reptiles from the late early Miocene (MN 4) of Béon 1 (Montréal-du-Gers, southwestern France). *Geodiversitas, 27*, 413–441.

Rage, J.-C. & Danilov, I.G. (2008) A new Miocene fauna of snakes from eastern Siberia, Russia: Was the snake fauna largely homogenous in Eurasia during the Miocene? *Comptes Rendus Palevol*, *7*, 383–390.

Rook, L., Delfino, M. & Sami, M. (2015) I vertebrati fossili della cava del Monticino di Brisighella: una finestra sui popolamenti continentali del Mediterraneo nel Miocene superiore. *Memorie dell'Istituto Italiano di Speleologia s. II, 28*, 79–100.

Szyndlar, Z. (1987) Snakes from the lower Miocene locality of Dolnice (Czechoslovakia). *Journal of Vertebrate Paleontology*, *7*, 55–71.

Szyndlar, Z. (1991) A review of neogene and quaternary snakes of Central and Eastern Europe. Part 11: natricinae, elapidae, viperidae. *Estudios geológicos, 47*, 237–266.

Szyndlar, Z. (2000) The snakes (Reptilia, Serpentes) of the Miocene of Portugal. *Ciencias da Terra, 14*, 359–364.

Szyndlar, Z. (2009) Snake fauna (Reptilia: Serpentes) from the Early/Middle Miocene of Sandelzhausen and Rothenstein 13 (Germany). *Paläontologische Zeitschrift, 83*, 55–66.

Szyndlar, Z. & Rage, J.-C. (2002) Fossil record of the true vipers. *In:* Schuett, G.W., Hoggren, M. & Douglas, M.E. (Eds), *Biology of the vipers*. Eagle Mountain Publishing, Eagle Mountain, pp. 419–444.

Venczel, M. (1998) Late Miocene snakes (Reptilia: Serpentes) from Polgárdi (Hungary): a second contribution. *Acta Zoologica Cracoviensia*, *41*, 1–22.

Venczel, M. (2011) Middle-late Miocene snakes from the Pannonian Basin. *Acta Palaeontologica Romaniae, 7*, 343–349.

Venczel, M. & Stiuca, E. (2008) Late middle Miocene amphibians and squamate reptiles from Taut, Romania. *Geodiversitas*, *30*, 731–763.

Wüster, W., L. Peppin, C. E. Pook, and D. E. Walker. 2008. A nesting of vipers: phylogeny and historical biogeography of the Viperidae (Squamata: Serpentes). Mol. Phylogen. Evol. 49:445–459.

Zerova, G. (1993) Late Cainozoic localities of snakes and lizards of Ukraine. *Revue de Paléobiologie, 7*, 273–280.
